# ETL: From the German Health Data Lab data formats to the OMOP Common Data Model

**DOI:** 10.1371/journal.pone.0311511

**Published:** 2025-01-06

**Authors:** Melissa Finster, Maxim Moinat, Elham Taghizadeh

**Affiliations:** 1 Fraunhofer Institute for Digital Medicine MEVIS, Bremen, Bremen, Germany; 2 Erasmus University Medical Center, Rotterdam, South Holland, Netherlands; Politecnico di Milano, ITALY

## Abstract

**Objective:**

The German Health Data Lab is going to provide access to German statutory health insurance claims data ranging from 2009 to the present for research purposes. Due to evolving data formats within the German Health Data Lab, there is a need to standardize this data into a Common Data Model to facilitate collaborative health research and minimize the need for researchers to adapt to multiple data formats. For this purpose we selected transforming the data to the Observational Medical Outcomes Partnership Common Data Model.

**Methods:**

We developed an Extract, Transform, and Load (ETL) pipeline for two distinct German Health Data Lab data formats: Format 1 (2009-2016) and Format 3 (2019 onwards). Due to the identical format structure of Format 1 and Format 2 (2017 -2018), the ETL pipeline of Format 1 can be applied on Format 2 as well. Our ETL process, supported by Observational Health Data Sciences and Informatics tools, includes specification development, SQL skeleton creation, and concept mapping. We detail the process characteristics and present a quality assessment that includes field coverage and concept mapping accuracy using example data.

**Results:**

For Format 1, we achieved a field coverage of 92.7%. The Data Quality Dashboard showed 100.0% conformance and 80.6% completeness, although plausibility checks were disabled. The mapping coverage for the Condition domain was low at 18.3% due to invalid codes and missing mappings in the provided example data. For Format 3, the field coverage was 86.2%, with Data Quality Dashboard reporting 99.3% conformance and 75.9% completeness. The Procedure domain had very low mapping coverage (2.2%) due to the use of mocked data and unmapped local concepts The Condition domain results with 99.8% of unique codes mapped. The absence of real data limits the comprehensive assessment of quality.

**Conclusion:**

The ETL process effectively transforms the data with high field coverage and conformance. It simplifies data utilization for German Health Data Lab users and enhances the use of OHDSI analysis tools. This initiative represents a significant step towards facilitating cross-border research in Europe by providing publicly available, standardized ETL processes (https://github.com/FraunhoferMEVIS/ETLfromHDLtoOMOP) and evaluations of their performance.

## Introduction

The expansion of healthcare research beyond institutional boundaries offers new opportunities, such as rapidly establishing a reliable decision-making foundation during pandemics or studying rare diseases [1–3]. However, these opportunities are accompanied by challenges in data security, protection, and the use of various representations and terminologies. By adopting a Common Data Model (CDM), these challenges can be addressed, enabling the utilization of federated networks to execute the same analytical pipeline across multiple data sources. Among the various available CDMs, the Observational Medical Outcomes Partnership (OMOP) CDM stands out due to its widespread adoption, ability to represent diverse data sources, and the availability of software tools that support the Extract, Transform, and Load (ETL) development process, quality assessment, and analysis [4].

In Europe, the establishment of Health Data Hubs, such as those in Finland, France, and Germany [5–7], is becoming increasingly prevalent. The French Health Data Hub is one of the 187 data partners of the European Health Data and Evidence Network (EHDEN) [8], setting an exemplary model for cross-border research. The German Health Data Lab (HDL) is currently under development. The HDL aims to maintain pseudonymized claims data from approximately 90% of German citizens insured within the statutory health system [9]. The claims data is collected annually by the National Association of Statutory Health Insurance Funds (GKV-SV) and transferred to the HDL [9]. Additionally, German citizens are going to have the opportunity to contribute their electronic health records (ePA) to enhance health and care. Over the years, the data scope and format have undergone changes, making it challenging for researchers to address research questions that require longitudinal data. The German Federal Ministry of Education and Research supports many German and European projects to drive health data harmonization forward, *e.g.,* the Medical Informatics Initiative and Real4Reg [10–12].

To combine HDL data from multiple years, an adaptation from one format to another is necessary. However, with the vision of European interoperability and reusability [13], we strive for a representation in a CDM. Popular CDMs are, for instance, the PCORnet CDM, Sentinel CDM, i2b2 CDM and OMOP CDM. We evaluated the CDMs against the literature and the characteristics of the source data to conclude that the OMOP CDM is the best fit [14–16]. To represent the HDL data in the OMOP CDM, we developed an ETL process. An ETL converts a source data model into a target data model by transforming its structure and semantics into the target structure and semantics [17]. The adoption of a common representation greatly facilitates the familiarization and analysis of HDL data. Numerous projects aiming to transform health data into the OMOP CDM are already underway [8, 12, 18, 19].

In Section Input Data we describe the data structure of the HDL formats 1 and 3, as well as their characteristics. We provide a short introduction to the OMOP CDM and used Observational Health Data Sciences and Informatics (OHDSI) tools in Section Introduction to OMOP CDM. In Section ETL Pipeline we visualize the workflow of the ETL process, explain the ETL process and local concepts in Subsection ETL Documentation and Local Concepts. We expand on the ETL creation, its details, and challenges specific to each format in Subsection Transformation of 45 Format 1, and Subsection Transformation of Format 3. Additionally, a quality assessment of both ETL processes is described in Section Quality Assessment. Finally, we take a closer look into the results in Section Results and discuss them in Section Discussion. Finally, we illuminate the limitations and conclude our work.

## Materials and methods

### Input data

Over the years, the scope and format of the transmitted claims data have undergone changes. The format is defined by its structure specified in the data dictionary. The earliest format, referred to as Format 1, hosts data from 2009 to 2016. Format 2, covering data from 2016 to 2018, is structurally equal to Format 1 but without applying risk structure compensation (RSC). RSC addresses disparities, such as those in salary and health of insured parties, between health insurances [20]. The RSC is applied solely for financial reasons. Since Format 1 and Format 2 have the same structure and the ETL process does not take the RSC into account, the same ETL can be applied. The third format, Format 3, includes data from 2019 onwards. The data scope is determined by law and polished by GKV-SV [21]. Detailed format descriptions can be found in the HDL repository [22].

Since real data cannot be accessed yet, example data was set up on a PostgreSQL Server. The example data used for the ETL development was provided by the HDL. The example data is randomly generated and lacks any realistic distributions. The example data, adhering to the structure of Format 1, is referred to as example data 1, while example data for Format 3 is called example data 3. The HDL does not provide example data for Format 2, since it matches the structure of Format 1. Example data 1 contains 3,432,000 persons per year, and example data 3 contains 100,001 persons per year.

Since the methods, scripts, and quality assessments were entirely developed using fictional data, this research does not involve any human participants or tissues. Hence, no ethical approval was required for this study.

#### Format 1 and Format 2

Formats 1 and 2 share a common ETL process due to their equal data structure. The claims data in Format 1 is organized into 9 tables:

- Insured person demographical information for the compensation year
- Insured person RSC (Reduced-earning-capacity pension and reimbursement of insured days) from the previous year
- Extracorporeal blood purification
- Outpatient drugs
- Inpatient diagnoses
- Outpatient diagnoses
- Expenditures
- Insurance Membership
- Municipality code

**Fig 1:**
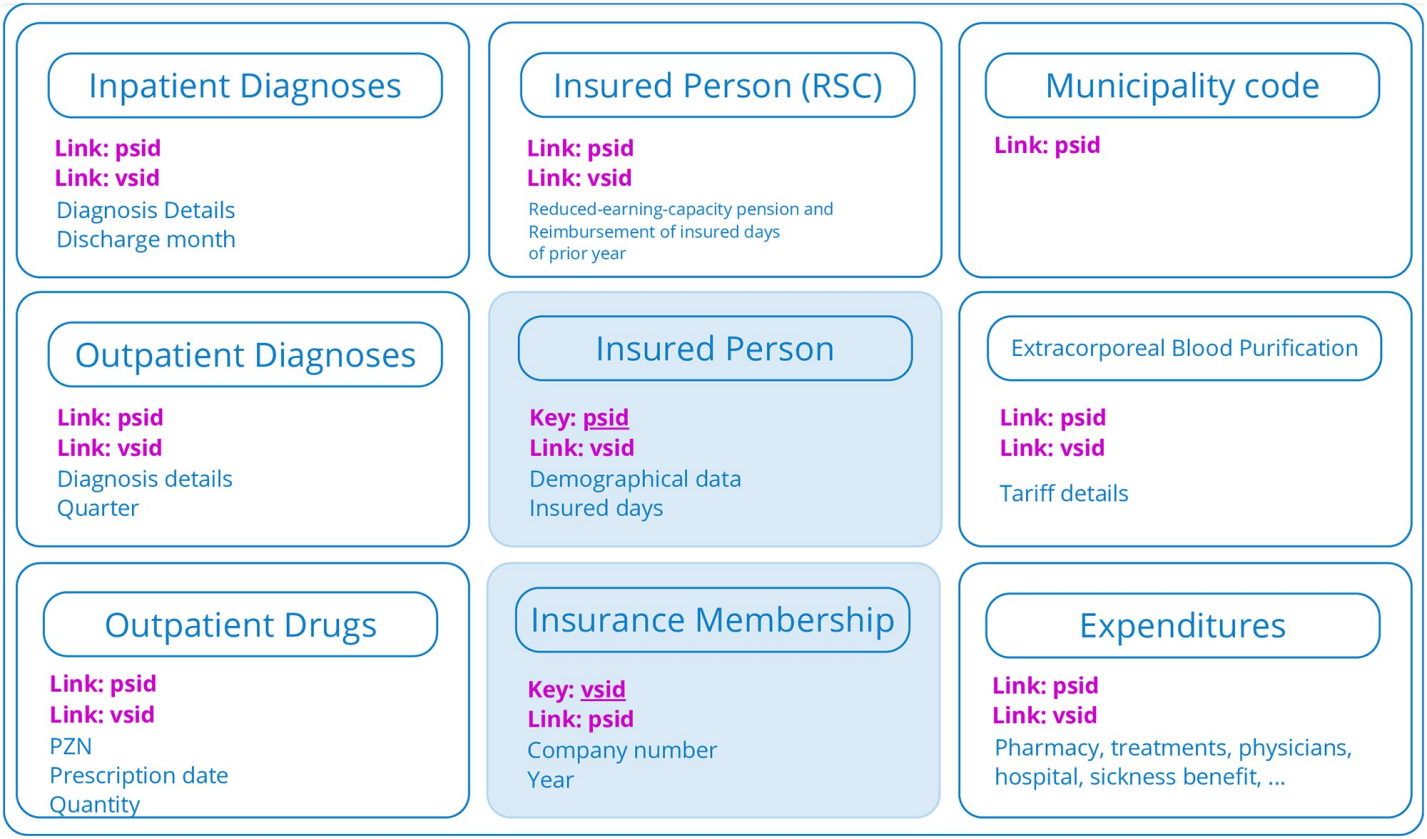
Visualization of Format 1. Format 1 with its primary keys, the long-term insured person pseudonym *psid* and short-term *vsid*.

A visualization of Format 1 is shown in Fig 1. One particularity is the distinction between the reported year and the compensation year. The reported year represents the year in which the data was collected, and expenses were incurred. The data is always transformed with respect to the reported year. Tables are interconnected using pseudonymized primary and foreign keys. They cannot be associated with an individuals identity. Format 1 has two identifiers (IDs): *psid*, which remains constant for an insured person over the years, and *vsid*, which changes annually and when a person changes health insurance. Dates are mostly vague, posing challenges for the transformation into OMOP and requiring several assumptions. For example, outpatient diagnoses are reported by the quarter of the year, inpatient diagnoses by the discharge month, and prescriptions by the specific date. For all other tables, only the year of the event is given. Apart from extracorporeal blood purification, no procedure codes are transmitted. Diagnoses are encoded using the German modification of the 10th revision of the International Statistical Classification of Diseases and Related Health Problems (ICD-10-GM), and medical products are encoded using the Pharmazentralnummer (PZN), a German identification catalog for drugs and pharmacy products.

#### Format 3

**Fig 2:**
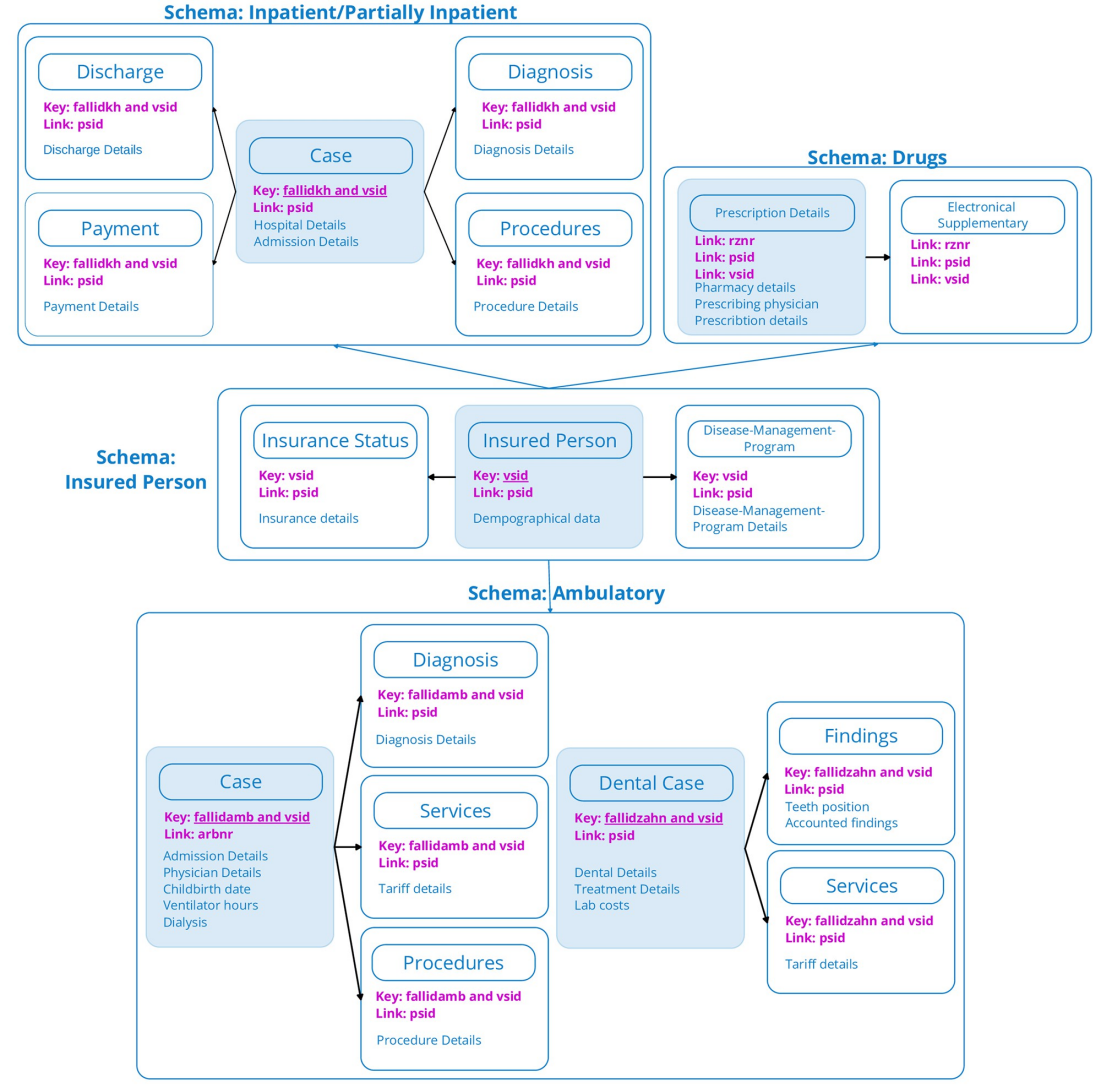
Visualization of Format 3. The four schemas of Format 3, with the universal key *psid* serving as the long-term pseudonym for the insured person, *vsid* the year and health insurance dependent key and schema-specific non-unique keys.

The current data format, referred to as Format 3, covers data from 2019 onwards. It encompasses a significantly wider scope, including more details as can be seen in Fig 2. Fortunately, date details are mostly provided with specific days. Many fields in Format 3 are optional, for instance several date fields. We transform the most accurate data available. If the precise date is not available, more general data is used. For example, for a visit to the dentist, we first check for a treatment date; if not available, we check whether a quarter is specified. If neither is available, the year is used. As the dates are in different source tables, this approach leads to additional join operations. Unlike Format 1, Format 3 features many scheme specific IDs. Each insured person is assigned a pseudonymized ID called *psid* and a year and health insurance dependent ID, called *vsid*. Additionally, identifiers exist for each schema. The scheme specific IDs can be uniquely assigned by the help of the *vsid*. The scope of Format 3 is broader, providing more comprehensive billing information.

The data in Format 3 is divided into four schemas:

- **Insured person** contains tables with demographic information, such as birth year and vital status, insurance relationships, and disease management programs.
- **Drugs** includes tables related to prescribed drugs, encompassing their PZN, quantity, prescription day, costs, pharmacy information, and prescribing physician and their facility.
- **Ambulatory cases** covers all outpatient cases, including dental cases. Each case is assigned to an ID, linking diagnoses, services, procedures, and all related information. Details include information about the treating and transferring physicians, facility, health insurance, first treatment day, last treatment day, service time, and tariff number.
- **Inpatient and partially inpatient cases** contain detailed information about admitted hospitals, departments, admission reasons, admission and discharge dates, discharge reasons, diagnoses, and procedures. The partially inpatient cases refer to regular daily stays in a facility without overnight stays. Diagnoses are coded using ICD-10-GM, while procedures are coded using the German Operation and Procedure Code (OPS) [23].

### Introduction to OMOP CDM

The OMOP CDM is a patient-centered and domain-oriented common data model. OHDSI supports several database management systems. The OMOP CDM has the capability to integrate data from various sources such as electronic health records (EHR), claims data, survey data, notes, oncology, and genomic variants [24–26]. The OMOP CDM is maintained by the OHDSI community, which originated from OMOP in 2014 [27].

The observational period table defines the period during which we expect healthcare events for a person to be captured, while the visit occurrence table contains information about the healthcare encounters, such as hospital stays. Clinical events can be linked to a visit occurrence. For semantic interoperability, standard concepts from the OMOP vocabularies are used to represent the clinical facts. For instance, SNOMED is used for diagnoses [28]. This requires developers to map their source codes to the OMOP standard concepts while retaining the original concepts in the provided source fields. ATHENA (Automated Terminology Harmonization, Extraction and Normalization for Analytics) provides freely available mappings of commonly used vocabularies to the standard concepts [29, 30].

OHDSI provides several tools to support, e.g., ETL development, quality assessment, cohort selection, or analysis [24, 26, 31–35]. We briefly introduce OHDSI tools, which are used for developing our ETL pipeline.

**White Rabbit** scans the source data to generate a file containing the format structure and data characteristics [36].

**Rabbit in a Hat** is used to support the creation of ETL documentation and generating a Structured Query Language (SQL) skeleton. Although the SQL-Skeleton does not include any logic mappings, it provides a convenient starting place for the ETL, with source-to-target references [37].

**Usagi** is used to create mappings from local vocabulary to the standard concept. Local codes, such as professional specialization codes for physicians, are loaded as a comma-separated values (CSV) file into the tool. Usagi suggests the best fitting standard concepts, chosen by string similarity.

**Data Quality Dashboard (DQD)** is used to assess the quality of the data mapped to the OMOP CDM and runs a standard set of over 4000 checks in the categories of Plausibility, Conformance, and Completeness [35, 38].

**Achilles** is used to compute aggregated statistics which can be used to visualize the characteristics of the transformed data [35, 39].

**CDM Inspection** creates an overview of the Achilles results in a Microsoft Word document [40].

### ETL pipeline

**Fig 3:**
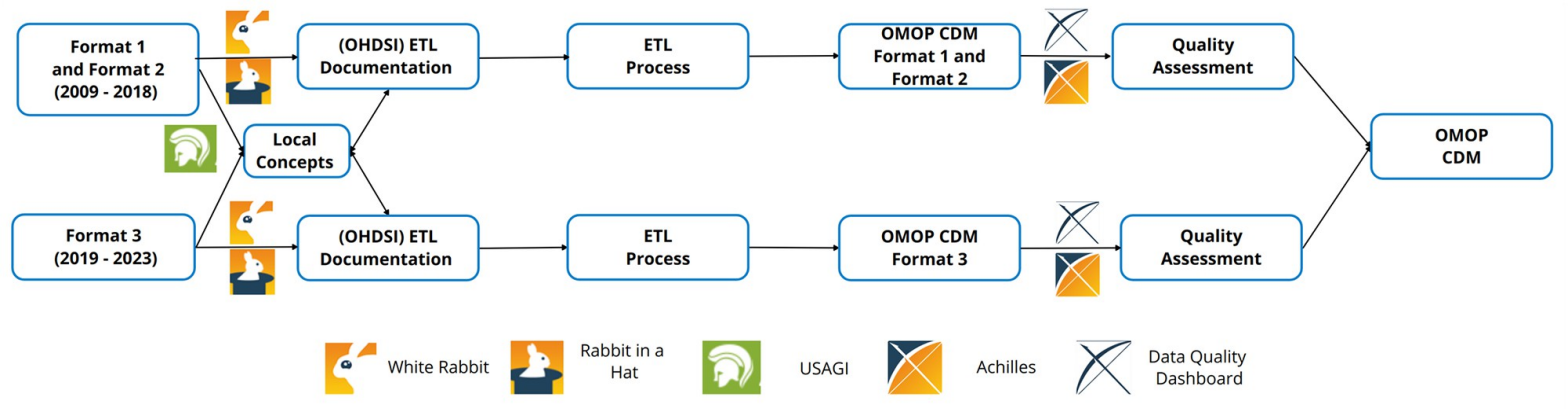
The ETL process from the original formats to the OMOP CDM. OHDSI tools were used to create local concepts, ETL Documentations, and Quality Assessment. Local concepts and ETL documentation were developed in parallel in order to incorporate progress.

Fig 3 depicts the workflow from the original formats to the OMOP CDM. In this work, we utilized OMOP CDM version 5.4 by employing the Data Definition Language SQL scripts for PostgreSQL from the OHDSI repository, and incorporated all concepts from ATHENA recommended for OMOP CDM version 5.4 [25, 35]. Additionally, we included the mappings for ICD-10-GM and OPS available on ATHENA. We use the vocabulary version v5.0 23-JAN-23. The same approach and tools are utilized for both formats, albeit implemented twice, once for Format 1 and once for Format 3. Data from Format 2 undergoes the same process as example data 1. As no data in Format 2 is available, the process is not discussed in detail.

#### ETL documentation and local concepts

Both example data sets were scanned with White Rabbit. With Rabbit in a Hat, we created ETL documentations and generated SQL skeletons. We developed local concepts for Format 1 and Format 3, with the help of Usagi. The suggested mapping was accepted without manual verification if the string similarity was 100%. In all other cases, we manually verified the suggested mapping to ensure its accuracy. Mapped local concepts encompass specialist categories, disease management programs, a broad classification of dental procedures, and insurance claims. Given that the source concepts are quite general and do not involve any medical classification codes, the authors could perform the mapping without needing an extensive medical background. For instance, the *COPD disease management program* was mapped to *Management of chronic obstructive pulmonary disease*. Since Format 3 is an extension of Format 1, we reuse the local concepts of Format 1 and complemented them with the missing terms of Format 3.

#### ETL process

In Subsection Transformation of 45 Format 1 and Subsection Transformation of Format 3, we provide detailed explanations of our implemented SQL queries. To handle data of more than one year, updates of the person and observation period table are implemented. To avoid conflicts, logical constraints are implemented. The final SQL queries are wrapped into a python program, running the whole ETL process. Constraints and key settings are executed at the end of the ETL process to optimize efficiency. To fulfill the constraints, some date-related assumptions must be made. They are discussed in the following subsections. For easy execution, the ETL processes for Format 1 and Format 3 are implemented as two independent Docker services. They can run in parallel. If data from more than one year of a format is provided, it is transformed sequentially.

#### Transformation of Format 1

**Fig 4:**
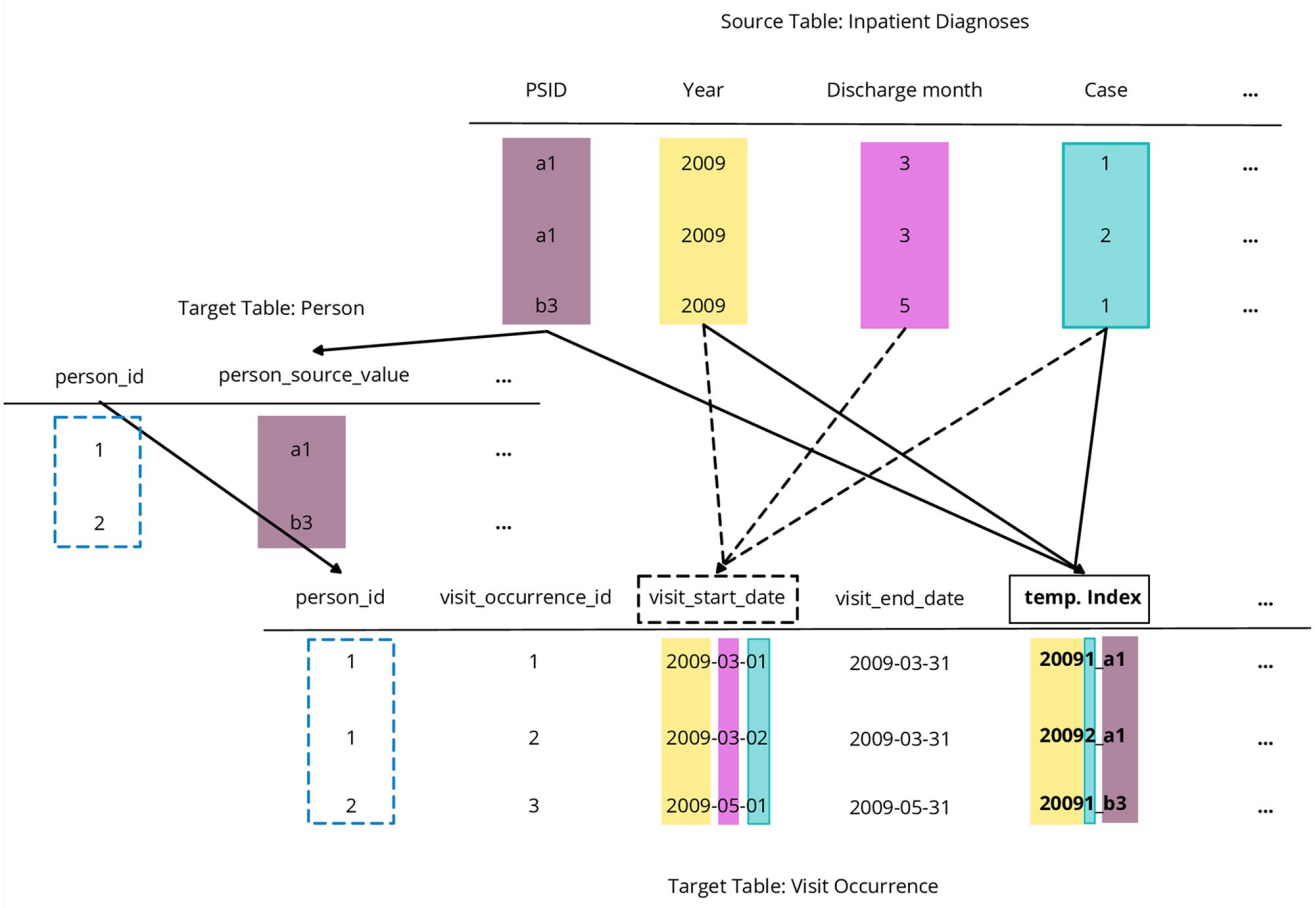
Snippet of the transformation of Format 1 to OMOP CDM. The temporary index is generated with the help of *psid, year, case* of the source table *Inpatient Diagnoses* to ensure unique event assignments. The case number is considered within the *visit_start_date* to keep the order of the diagnoses without knowing the specific date of the cases.

Firstly, the Person table is created. Since the source primary key (psid) does not conform to the numerical key convention of the OMOP CDM, we generate unique person IDs in the OMOP CDM person table. We store the *psid* as source value and use it as a lookup table. Many individuals are expected to appear annually in the HDL data. Therefore, we update the *person* table whenever a *source_value* matches the *psid*. Since Format 1 only utilizes *psid* and *vsid* as primary keys (see Fig 1), it is necessary to execute the transformation queries in a specific order to establish links between the OMOP CDM tables. For example, we map information from the source table *inpatient cases* to the target tables of *visit occurrence* and *condition occurrence*. Since the source table does not have a primary key, except the tables *Insured Person* *(psid)* and *Insurance Membership* *(vsid)*, we add a temporary key in the visit occurrence table to ensure the correct assignment of the *visit occurrence* ID to the *condition occurrence*. The data is transformed first into the visit occurrence table, linked by the person table to get the person ID; see Fig 4, and finally the diagnosis of the source table *Inpatient diagnosis* is transformed into the condition occurrence table.

We automatically generate new primary keys for all tables, except for the payer plan period id, for which we utilize the *vsid*.

*Date and period assumptions*. As mentioned in Section Input Data, the provided date information lacks granularity. Biedermann and colleagues suggest to extract dates from related events [41]. In instances where additional date information cannot be obtained, they propose setting the date to the first day of the month, quarter, or year, specifically for drug-related cases. We have adopted this suggestion for other scenarios as well. OHDSI provides guidance for handling most mandatory date fields, such as the day of death [25]. We have adhered to OHDSI’s guidelines. Therefore, we consider the first day of the given period as the event date for all events, except for inpatient and partially inpatient cases as well as for the day of death. For example, if the discharge date is specified only as the second quarter, we assume that the admission occurred on April 1st. As first exception of the rule, we consider the case enumerator, provided in the source data of inpatient and partially inpatient diagnosis; see Fig 4. To keep the order of the diagnoses we use the same approach as for the other events but add one day for each following case. The second exception to this rule is the date of death. Since only the year of death is known, we set the day of death to December 31st. By making these assumptions, we ensure that no events are excluded.

For each person in the data, we create an observation period of one year, assuming that they were insured continuously throughout the entire year. Although we have information on the number of insured days within an insurance, we do not have access to the start and end dates of the insurance coverage. This can lead to contradictions if a citizen was insured by multiple statutory health insurances and at least one private health insurance. For insured individuals without any visits, determining the order of insurance relationships is impossible even with complex queries. As a result, we assign a one-year insurance period to each insurance relationship. The correct insurance can still be identified through its *vsid*. The number of days with reduced earning capacity and reimbursed insurance days are recorded as observations using a local concept. If an observation period for a person already exists, we simply extend the period.

*Diagnosis*. The *stationary diagnosis* and *ambulatory diagnosis* tables have similarities. Both tables create one entry in the OMOP CDM table visit occurrence with the corresponding visit occurrence ID and one or more entries per source row, depending on the target domain, in procedure, condition, observation occurrence, or measurement table. Although all ICD-10-GM codes are collected in the diagnosis source table, in the OMOP CDM, an ICD-10-GM code can belong to a condition, procedure, observation, or measurement. The domain of OMOP’s concept determines the tables to which the diagnoses belong. To create this relationship, we utilized a SQL-view and leveraged the ICD-10-GM mapping available on ATHENA.

We use the visit occurrence ID to link the visit to its diagnosis-related tables. Additionally, stationary diagnosis distinguishes between Inpatient, Outpatient in Hospital, and ambulatory clinic/center which can be directly be mapped to the visit concept.

Ambulatory diagnoses of the Format 1 are handled in the same manner as stationary diagnoses. Additionally, we classified diagnoses as preliminary, resolved, confirmed, or excluded diagnoses. We map the excluded diagnoses to the observation table and identify them with the concept ID *Disorder excluded*. All other classifications can be mapped to the status concept ID of the corresponding occurrence table. The ICD-10-GM code mapping is carried out similarly to the stationary diagnoses.

*Procedures*. In Format 1, procedure codes are not provided, but a blood purification table is present. We map Extracorporeal dialysis to the SNOMED concept Dialysis procedure, which is more general than the original information. Since neither the date nor the number of blood purification sessions is known, we include only one session dated on the 1st of January.

*Drugs*. Prescriptions are only available as PZN code. There is no publicly available mapping from PZN to RxNorm yet. Due to licenses, restriction on time and experts capacity an in-house mapping was not feasible. Therefore, we map it to standard concept “0” and retain the PZN as the source value.

We only have access to the prescription date, and no information on the start and end date, duration, or ingestion of intake. Since the duration of the medication is not available, we use the OHDSI convention, *i.e.,* we add 29 days to the start date [25].

*Costs*. It is important to consider the primary use of claims data, which is billing information collected by health insurances. We capture this information in the cost table, which is based on the US health system. Consequently, the HDL data align only partially with the structure of the OMOP CDM cost table. However, we can still include the amount and cost type, such as dental claim or hospital costs. Furthermore, with the payer plan period id, we can establish links to the health insurance and the insured period.

#### Transformation of Format 3

Format 3 is more comprehensive than Format 1. The four schemes within Format 3 are independent of each other, so we used them as a trivial separator. Data from the four source schemes are converted into the OMOP CDM with one entry in the person table and one observation period per insured person. If an existing *psid* appears again, which occurs for most insured individuals annually, we apply an update to the person table. In accordance with Format 1, we extend the observation period. The tables in Format 3 mostly provide day-specific date information, sometimes even timestamps. If that is not the case, we narrow it down to a quarter and use the first day of the quarter if available, similar to Format 1. All schemes provide a person ID *psid*, an insurance ID *vsid* and each schema has an additional case ID that enables linking between tables within a schema. To utilize all available information, we create local concepts for key tables, which are accessible through GKV-SV [42]. Format 3 does not have a summarized table for care site and provider. Therefore, for each new care site and provider, we create an additional entry in the respective tables and map as many details as possible.

*Insured person*. The first schema, *insured person*, includes tables such as death, location, procedure occurrence, observation period, and observation. The OMOP CDM table person only supports mapping binary genders (male and female). In this data format, the day of death is provided, and the observation period can be narrowed down to quarters. However, we want only one observation period in case the person is insured for several quarters in a row. We check for the first and last quarter in which the person appears and generate one observation period per health insurance per year, accordingly. For example, if records for an individual are found starting in the first quarter and ending in the third, we assume the person was also insured in the second quarter. The used insured days are mapped similarly to the insured days of Format 1, using the complemented local concept from Format 1. Disease management programs are mapped to similar procedure codes, if available.

*Stationary and partial stationary hospital cases*. The schema *stationary and partial stationary hospital cases* contains case information such as admission and discharge dates, care site and physician information, as well as diagnoses, procedures, and payments. In the observation table, we keep the reason for admission using a local concept. In the visit occurrence table, we capture the reasons for admission and discharge, as well as the type of stay. We use details from two source tables to generate one complete entry in the visit occurrence table.

The diagnoses codes of partial stationary and stationary hospital visits are mapped as described in the ETL process of Format 1, but we additionally distinguish between admission, primary, and secondary diagnoses. These codes are originally stored in the table *khdiag* without dates. To determine a start date and end date, we use the case table in addition. Moreover, we link the target entry to a visit.

Procedures are coded using OPS codes, and we utilize the available mapping from ATHENA to map these procedures to the standard concept. The location information, such as left, right, or both sides, is only kept as source value. Like diagnoses, the OPS code might belong to a different domain than in the source. Therefore, we use a similar approach as for the diagnoses codes. Depending on the domain, OPS codes might be mapped to procedures, observations, measurements, or drugs. The entry is linked to a visit. Stationary and partial stationary visits also include payment information, which is mapped to the cost table with a link to the corresponding visit entry.

*Ambulatory cases*. The *ambulatory scheme* is further divided into two themes: general claims and dental claims. Cases in the ambulatory scheme can only be linked to the respective claim. For general claims, mappings to visit occurrence are handled similarly to hospital cases. If a visit is caused by an accident, we capture this information as an observation. ICD-10-GM diagnoses with special characters are kept as source value and mapped to the standard concept without the special characters and location. As for Format 1, excluded diagnoses are only mapped to the observation table with the observation concept ID *Disorder excluded*. OPS codes are mapped to procedures, observations, measurements, or drugs, depending on the target domain. We followed the OHDSI convention by using the start date as the end date when the end date is not available but required [25]. Additionally, blood purification can be mapped with day-specific date information. Furthermore, we map the tariff number to procedures. The cost table contains the total charge based on the tariff number.

The source table *dental case* has its own case number and is treated differently from general claims in the source scheme *ambulatory*. Since the start day of a dental visit is not a mandatory field, we check if a date is provided; if not, we use the first day of the quarter. If the end date is not provided, we assume that the visit lasted no longer than one day and use the start date as the end date. The type of treatment, such as early treatment or regular treatment, is kept in the source value of the visit occurrence table since there is no fitting standard concept. Detailed teeth information is not stored, but we keep the findings as source value in the observation table. Dental services are mapped to procedures. They are not OPS-coded but categorized into five groups.

We directly map these groups to standard concepts and retain the original value as source value. The procedure information is given by the tariff number of the German Uniform Assessment Standard (UAS) [43], which is primarily used for charge calculation. We utilize the mapping provided by TU Dresden to retain the information [44]. All dental costs, including case costs, lab costs, and external lab costs, are stored as a sum in the cost table, linked to the visit, and marked as a dental claim. Currently, the OMOP vocabularies do not contain standard concept for the position of teeth. Therefore, we acknowledge the loss of some dental details in the transformation process.

*Drugs*. The *drugs schema*, containing prescribed drugs, are mapped to the drug exposure table. As for Format 1, drugs are coded in PZN and therefore map only to standard concept ID 0. Pharmacy costs are linked to the payer plan period and split into *paid by patient*, *total costs*, and *total charge*.

### Quality assessment

During the development phase we continuously checked random transformations of patients against the expected outcome. While this approach does not provide an overall assessment, it is a crucial step. We compare our expectations with the ETL results, considering that one column in the source data may translate into multiple columns in the target model. This check requires a deep understanding of both the source format and the OMOP CDM and cannot be fully covered by general analyses.

However, we evaluate the quality of the ETL process by employing the following tools and methods on the converted example data:

- **Field coverage of transformation** describes the ratio of transformed fields against the overall number of fields in the source data. To compute the field coverage we excluded unneeded fields, e.g., redundant information.
- **DQD** to verify and validate the Conformance and Completeness of the transformed data in the OMOP CDM. Since the example data set does not contain logical data that would provide meaningful results, we disabled the plausibility checks.
- **Achilles and CDM Inspection:** are used to give an overview of the returned results of the Achilles vocabulary mapping.

## Results

In this section we take a closer look at the results of the quality assessment and field coverage. As visualized in Fig 3, we analyze the two formats separately. The CDM Inspection results can be found in Supporting Information S1 File. CDM Inspection of HDL Format 1 and S2 File. CDM Inspection of HDL Format 3. Additionally, the comprehensive quality assessment results can be found in the repository (https://github.com/FraunhoferMEVIS/ETLfromHDLtoOMOP).

### Format 1

#### Field coverage of transformation

In Format 1, 38 fields are successfully transformed into the OMOP CDM, while seven fields are excluded. Out of these, four fields were deliberately omitted as they contained non-essential or redundant. Detailed explanations and lists are provided in Supporting Information S1 Table. Fields of Format 1 which were not intended to be kept. Table 1 outlines the fields that were intended for transformation but could not be included in the ETL process. They are briefly discussed in the Discussion. When considering only the fields intended for retention, the coverage rate achieved is 92.7%.

**Table 1:** Fields of Format 1 which cannot be transformed.

Field	Field Explanation	Reason
icd_zusatz	Additional special characters of ICD 10 code	No fitting target field
lokalisation	Localization of OPS code	Missing mapping
versichertentage	Insured days	Source lacks information

#### DQD

Table 2 displays the results of the DQD for Format 1. 99.9% of the conformance checks and 80.6% of the completeness checks pass in the verification step. In the validation step 100.0% conformance and 81.3% completeness is obtained.

**Table 2:** DQD results of Format 1.

	Verification				Validation			
	Pass	Fail	Total	% Pass	Pass	Fail	Total	% Pass
Conformance	925	1	926	99.9%	145	0	145	100.0%
Completeness	361	87	448	80.6%	13	3	16	81.3%
Total	1286	88	1374	93.6%	158	3	161	98.1%

#### Achilles and CDM inspection

The CDM Inspection provides an overview of the mapped concepts and its coverage of the ETL process. The number of unique codes (codes source, codes mapped) and the total number of codes with multiple occurrences (records source, records mapped) of Format 1 are displayed in Table 3.

**Table 3:** Code mapping coverage of Format 1. The Table comprises all OMOP CDM domains including local concepts and ICD-10-GM to standard mapping. Example data 1 contains some fictional ICD-10-GM codes. A PZN mapping to map codes from domain Drug to standard concepts is lacking.

Domain	Codes Source	Codes Mapped	% Codes Mapped	Records Source	Records Mapped	% Records Mapped
Condition	34,622	6,334	18.3%	12,422,894	2,421,782	19.5%
Condition status	6	5	83.3%	12,422,894	11,042,042	88.9%
Drug	3,432,000	0	0.0%	6,864,000	0	0.0%
Measurement	35	35	100.0%	12,342	12,342	100.0%
Observation	28,665	28,664	100.0%	35,758,734	1,438,734	4.0%
Procedure	79	79	100.0%	3,461,788	3,461,788	100.0%
Visit	3	2	66.7%	13,728,000	10,296,000	75.0%

#### ETL performance

The ETL process for example data 1 runs twice, sequentially; once for the year 2009 and once for the year 2010. The data lie on a PostgreSQL server hosted on a cluster with 2,000 MHz CPU and 65,536 MiB memory reserved for the execution. The Python scripts execute locally in a Docker container. Within the docker container two scripts are called: 1) One to create the OMOP CDM and load concepts, and 2) one to apply the ETL process and to run indices and constraints queries. Due to the size of data, we open and close new connections to the PostgreSQL server for each query. Time measurement shows that establishing the connection and send the query takes in total only seconds which can be neglected.

One year of example data 1 contains 3,432,000 unique patient ids, 9 tables with in summary 83 columns, 30,891,120 rows within one year; a total size of 1.74 GB. Since example data 1 contains two example years, a total size of 3.48 GB is load into the database. In Table 4 the execution time for example data 1 in minutes is presented. The first script, i.e. creating OMOP CDM and loading concepts, is taking most of the time, about 86.2 minutes, out of which only about 5 seconds are spent for creating the OMOP CDM. Consequently, the rest of the time (approx. 86.1 minutes) is spent loading concepts, which depends only on the OMOP concepts and local concepts. Hence, it is completely independent of the data and its size. Executing the ETL process took approximately 44 minutes for more than 6.8 Million insured persons. Running indices and constraints at last is a performance driven decision and takes less than 33 minutes for example data 1.

**Table 4:** Execution time of ETL process for example data 1.

Script	Execution Time in minutes
Create CDM and load concepts	86.2
ETL process	44.3
Indices and constraints	32.7
Total	163.2

### Format 3

#### Field coverage of transformation

In Format 3, 100 fields are transformed while 56 are not. Of the 56 fields, 40 are intentionally not transformed: 19 fields are irrelevant, 12 are omitted in favor of retaining other details, five added no value, and four contain no data. A detailed list of these excluded fields along with their explanations is available in Appendix S2 Table. *Fields of Format 3 which were not intended to be kept*. Table 5 enumerates the fields that could not be transformed. The achieved field coverage is 86.21%.

**Table 5:** Fields of Format 3 which cannot be transformed.

Field	Field Explanation	Reason
versstatus	Insurance status	Source lacking details
inansprartamb	Type of claim, e.g., Consultation	No fitting target field
tsvgart	German Appointment Service and Care Act (TSVG)	No fitting target field
tsvgarzt		
tsvgbsnrkv		
tsvgbsnrpseudo		
tsvgdat		
zweitmein	Second opinion identification	No fitting target field
zahn	Coded teeth position	No fitting target field
refart	Subsequent findings	No fitting target field
wirkstoffvo	Active ingredient regulation	No fitting target field
fa	Admitted department	No fitting target field
abrvondat	Accounting date	No fitting target field
abrbisdat		
aufnfa	Admitting specialist department	No fitting target field

#### DQD

In Table 6, the results for Conformance and Completeness are shown. In the verification step, 918 out of 926 conformance checks passed, and 344 out of 448 completeness checks were successful. In the validation step, all 145 conformance checks passed and 8 out of 16 validation checks passed.

**Table 6:** DQD results of Format 3.

	Verification				Validation			
	Pass	Fail	Total	% Pass	Pass	Fail	Total	% Pass
Conformance	918	8	926	99.1%	145	0	145	100.0%
Completeness	344	104	448	76.8%	8	8	16	50.0%
Total	1262	112	1374	91.9%	153	8	161	95.0%

#### Achilles and CDM inspection

Table 7 displays the code mapping coverage of whole OMOP CDM for the transformed example data 3, including the number of unique codes (source codes, mapped codes) and the total number of codes with multiple occurrences (source records, mapped records).

**Table 7:** Code mapping coverage of Format 3. It comprises all OMOP CDM domains including local concepts, and ICD-10-GM and OPS to standard mappings. Example data 3 contains fictional OPS codes. A PZN mapping to map codes from domain Drug to standard concepts is lacking.

Domain	Codes Source	Codes Mapped	% Codes Mapped	Records Source	Records Mapped	% Records Mapped
Condition	53,122	53,003	99.8%	4,985,719	4,979,108	99.9%
Condition status	4	4	100.0%	4,985,719	4,985,719	100.0%
Drug	12,726	148	1.2%	1,604,096	2,251	0.1%
Measurement	330	330	100.0%	4,509	4,509	100.0%
Observation	6,389,435	11,917	0.2%	8,052,039	1,093,191	13.6%
Procedure	1,504,196	32,684	2.2%	9,922,480	3,019,807	30.4%
Provider Specialty	132	101	76.5%	1,567,763	821,794	52.4%
Visit	173	172	99.4%	1,624,715	1,212,814	74.6%

#### ETL performance

The ETL process for example data 3 runs for the year 2019. The set up is the same as described for example data 1. Even though example data 3 contains a significantly smaller number of insured persons (100001) than example data 1, it has a size of 1.42 GB due to more details, tables (17) and fields (217 columns, 22,098,449 rows). Since creating the OMOP CDM and loading the concepts are data independent, it takes approximately the same time as for Format 1; see Table 8.

**Table 8:** Execution time of ETL process for example data 3.

Script	Execution Time in minutes
Create CDM and load concepts	87.8
ETL process	22.1
Indices and constraints	18.5
Total	128.4

## Discussion

### Discussion of Format 1 quality assessment results

#### Field coverage

In Appendix S1 Table. *Fields of Format 1 which were not intended to be kept* four fields are listed which we do not want to transform. The decision on which fields to drop was made by the authors. For certain fields, this decision is straightforward, as the information is either redundant or not relevant. For example, we prioritize the actual date of an event over the year it was reported. For more complex decisions, we consulted with EHDEN members at the Erasmus Medical Center to leverage their expertise. The information of the fields in Table 1 is lost or could only partially retained. The icd_zusatz contains special characters, i.e., daggers, asterisks, and exclamation marks, which provide more information of the code, like if it is a primary, secondary or optional code. This can be partially kept by another field *qualifizierung*. Due to missing start and end date of insurance membership in the source data, a reasonable transformation of the total number of insured days (versichertentage) within a health insurance is not applicable.

During this work, we faced challenges in mapping local vocabularies to OMOP CDM. This issue has been observed in multiple studies from several European countries including France, Austria, the UK, and Germany [45–50]. Among these studies, only the study in France aimed at overcoming this issue by mapping drug vocabularies such as CIP13 and UCD to RxNorm vocabulary using a two-step approach. They first map from local vocabulary to the ATC (Anatomical Therapeutic Chemical) classification, and then from ATC to RxNorm. While achieving a high mapping rate (95.62% of CIP13 codes), it resulted in the loss of detailed information about drug formulations and dose levels. This limitation arises because the ATC classification system only contains information about the active compound and not the detailed formulation or dosage. The efforts across these countries highlight a common theme: while mapping to standardized vocabularies like OMOP can enhance interoperability and data sharing, it also involves trade-offs, particularly the loss of certain detailed local information. Future efforts might benefit from developing better mapping strategies that can retain important local details while achieving the interoperability goal.

#### DQD

The one conformance failure of Format 1 (Table 2) is caused by the drug strength tables. Since we do not load our own concepts into this table and only load concepts available on ATHENA, we consider this failure negligible.

If we take a closer look into the failures of the Completeness, we detect only expected failures, like five failed checks of table location, due to missing demographic data, e.g., *ADDRESS_1*. We notice, that even though fields are optional in OMOP, checks fail if they are not filled. However, some failures are also caused by the lack of mappings to standard concepts, like a missing mapping from PZN to RxNorm. Local concepts map many source codes to the standard concept, although some source codes cannot be assigned to a standard concept, such as tariff numbers or reimbursement of insured days. Consequently, they are assigned to the standard concept ID “0”. Completeness checks for the percentage of persons that have at least one entry in the table measurement and procedure fail since Format 1 does neither provide procedures nor measurements.

#### Achilles and CDM inspection

The low mapping coverage of condition concepts in Format 1, presented in Table 3, is a result of non-existing, artificially created ICD-10-GM codes in the example data 1. For the Drug domain only PZN is given, which is why no mapping is available. The one concept of condition status and visit which cannot be mapped, is the value “0”. In case the source value did not fit into any category or was left empty, a “0” was inserted into the obligatory field.

### Discussion of Format 3 quality assessment results

#### Field coverage

Format 3 contains comprehensive data, not all of it is relevant for our use case, e.g., data for administration purpose only, like when the data was transmitted or when it was reported. The data contains several check sums that are required for internal processes. However, this information has no added value for research. Other fields, where the reason *empty* in Appendix S2 Table. *Fields of Format 3 which were not intended to be kept* is noted, are placeholders, not filled with data. We do not keep details of pharmacies, yet drug details are kept. For hospital and physician, only the treating instances and primary information are kept.

Two fields with health insurance details of Format 3 cannot be kept due to the lack of background information, as shown in Table 5. Due to the structure of the OMOP CDM not all information fits. One can argue that every detail can be kept as an observation in the source value field. However, it is a trade-off between keeping everything while losing its context and accepting some information loss, e.g., German Appointment Service and Care Act (TSVG) details. For some target fields, we had to decide which of the source information to map into, e.g., we lose some information regarding the admitted department (fa, aufnfa), and instead keep facility details and profession of the physician.

Finally, we must take into account that some information might be generalized during the transformation or mapping process.

#### DQD

Besides the conformance failure, we already described for Format 1, we report 5 failures in the conformance category, because event dates were not within a week of the visit period, see Table 6. These are caused by the example data, where dates were created randomly without respect to visit periods. One failed check is caused by empty country concepts of the location table of medical practice. Even though most visits are interior, we do not have any information regarding the location. The last failed check can be ignored, since it is caused by a valid standard concept of UK Biobank, which was not locally included.

The Completeness category results in 104 failures for the Format 3. Several failures are caused by empty fields which are optional and not given by the source data. Since more tables were populated for Format 3, more missing value analysis failed than for Format 1. Additionally, as for Format 1 the PZN mapping is missing and some local concepts like the tariff number cannot be mapped to the standard concepts, which are consequently mapped to concept ID “0”. Due to the nature of claims data the measurement table records only a few values, which causes another DQD failure.

#### Achilles and CDM inspection

In Table 7 we obtain a high coverage of mapped condition codes with 99.8%. Originally, all conditions are coded in ICD-10-GM. However, it should be underlined, that the ICD-10-GM code distribution is randomly distributed and does not reflect the distribution of a real data set. For Format 3 we perceive a drug coverage higher than zero. These results from OPS codes which were categorized as procedure in the source data but due to the OMOP standard concept recategorized into the domain Drug. The domain Observation hosts information which does not belong elsewhere. Additionally, local concepts are mainly applied on the domain Observation and the UAS concept is also applied to the domain Observation. The low percentage of mapped OPS codes of domain Procedure results from artificial and partially local OPS codes. The coverage of Provider Specialties with 76.5%, and a code mapping of the Domain Visit with 99.4% result from local mappings.

## Limitation

Researching with claims data provides many opportunities but also presents certain challenges. For example, while all prescriptions are available, we cannot be certain that the patient actually took the prescribed medication. During our ETL process, we assume that the prescribed medication was taken.

Transforming data from its original format to a new format requires certain compromises and may result in some information loss. As described in Section ETL Pipeline, excluded diagnoses are mapped as observation, since the OMOP CDM does not consider excluded diagnosis as condition occurrence. Transforming them as observations ensures that excluded diagnoses are not accidentally considered as diagnoses during analysis. However, by using the concept id for excluded diagnosis, searching for the excluded disease via its OMOP standard concept is not possible. Yet, it can be searched by its ICD-10-GM code which is kept as source value.

In the future, we aim to retain details such as the localization of procedures and conditions (left side, right side, both sides). Henke and colleagues have taken a first step towards this by introducing a mapping of localization [44]. Since we aim to map to the standard concept including localization whenever possible, we currently accept losing localization information to avoid additional entries that solely contain localization information.

During the development of the ETL process we were facing challenges because of the absence of real data. Due to mock data, we miss invalid and illegal data. Even if data fields are marked as obligatory, they might be empty. Dates might lie in the future or be out of range. We considered several cases to catch these issues, but without real data they remain limited.

A popular method to measure the quality of transformation, is the comparison of results of a use case. Since we could not access real data, we do not have this opportunity. Even a simple statistical use case is meaningless, since provided example data does not consider any realistic distribution. For this reason, we aim to optimize our ETL process on real data as soon as one can apply for access at the HDL.

Mappings to standard concepts of heavily used German-specific vocabulary, like PZN and UAS, must be elaborated to enable network-wide studies. Due to the license-based nature of the PZN to ATC mapping catalog, there is no mapping from PZN to RxNorm available. This not only poses a barrier for interoperability but also results in a lack of relevant information. We do not have any information regarding ingredients, package size, recommended duration of use, and other details, which leaves us with generalized assumptions during the ETL process.

Some information gets lost during the ETL process, since the OMOP CDM, especially the cost table, is tailored to the US healthcare system. Some adaptations of the OMOP CDM towards the Europe health system are needed to avoid generalization and trade-offs. Moreover, the OMOP CDM must be enhanced to keep dental information in a meaningful way.

## Conclusion

An ETL process for Format 1 and Format 3 of the HDL data was developed and published (https://github.com/FraunhoferMEVIS/ETLfromHDLtoOMOP). A common representation eases the familiarization with the data and facilitates HDL users to execute one analysis of claims data represented originally in various formats. By developing the ETL process, being transparent on assumptions and transformation steps, evaluating its performance and making the scripts public available we have taken an important step towards collaborative health research. The scripts are designed to independently transform data for both Format 1 and Format 3. Therefore, we have not included a unified target schema. However, we have applied the union step to our example data.

To address the aforementioned limitations, we plan to execute our developed ETL process on real data. Initial steps will commence once the HDL is online. We aim to conduct an analysis using both the original data format and the transformed data to compare and analyze its outcome. An additional valuable step could be the integration of the ETL process within the HDL. To overcome mapping challenges, we aim to share our findings to advance publicly available PZN and UAS mappings.

## Supporting information

S1 Table Fields of Format 1 which were not intended to be kept. (PDF)

S2 Table Fields of Format 3 which were not intended to be kept. (PDF)

S1 File CDM inspection of HDL Format 1. (PDF)

S2 File CDM inspection of HDL Format 3. (PDF)
