# Supplementary material for: ETL: From the German Health Data Lab data formats to the OMOP Common Data Model
Source: PLoS One. 2025 Jan 6;20(1):e0311511. doi: 10.1371/journal.pone.0311511 (PMC11703056; doi:10.1371/journal.pone.0311511)
Supplement: S1 Table — (PDF) [file pone.0311511.s001.pdf]

Field	Field Explanation	Reason
ausgleichsjahr	Reported year of RSC	Not of interest
satzart	Table details	No added value
icd_code	ICD-10-GM code without separator, special characters	No added value
kv_nr_kennzeichen	Insurance indicator	Not of interest\not available
