# Supplementary material for: ETL: From the German Health Data Lab data formats to the OMOP Common Data Model
Source: PLoS One. 2025 Jan 6;20(1):e0311511. doi: 10.1371/journal.pone.0311511 (PMC11703056; doi:10.1371/journal.pone.0311511)
Supplement: S2 Table — (PDF) [file pone.0311511.s002.pdf]

Field	Field Explanation	Reason
bjahr	Reported year	Not of interest
version	data version	Not of interest
erstzeit	Creation time	Not of interest
bnr	Operation number	Not of interest
lfdnr	Serial number	Not of interest
nbsnrpseudo<br>nbsnrkv	Secondary premises pseudonym identifier	Keep only information of primary premises
sachkobez	Material cost designation	No added value
abrbegr	Settlement justification	No added value
bsnruebpseudo<br>bsnruebkv<br>lanruebpseudo<br>lanruebfg<br>lanruebpruef	Details referring physician	Keep only details of treating physician
befnrzahl	Number of accounted findings	Keep only cost and finding
vodat	Date of prescription	Keep pick-up day instead
vertragskz	Contract indicator (insurance, pharmacy)	No added value
apoklass<br>apopruef<br>apopseudo<br>aporegknz<br>apositz<br>apotyp	Pharmacy details	Not of interest
noctu	Free of charge pick-up in the night	Not of interest
autidem	If substitution allowed	Not of interest
zuzahl	placeholder	Empty
mehrkosten	placeholder	Empty
zaehler	placeholder	Empty
einheit	placeholder	Empty
zuzahlkz	Additional payment obligation	Not of interest
bsnrvovb	Supply sector of prescribing premises	Not of interest
lenrvopseudoarztnr	Verification number in tax table	Not of interest
faktor<br>faktorkennzeichen	Factor<br>Factor indicator	Keep prescribed amount instead of factor
khpruef	Verification	Not of interest
einweispruef	Verification	Not of interest
veranlasskhpruef	Verification	Not of interest
veranlasskhregknz	Regional identifier of arranging hospital	Keep only information of treating hospital
tageobe	Days without treatment	No added value
lenrvopruef	Verification	No added value
