# Supplementary material for: ETL: From the German Health Data Lab data formats to the OMOP Common Data Model
Source: PLoS One. 2025 Jan 6;20(1):e0311511. doi: 10.1371/journal.pone.0311511 (PMC11703056; doi:10.1371/journal.pone.0311511)
Supplement: S1 File — (PDF) [file pone.0311511.s003.pdf]

CDM Inspection report for the fdzformatnew database

Package Version: 1.2.4

Date: Wed Sep 4 16:09:53 2024

Authors: mfinster

This project has received funding from the Innovative Medicines Initiative 2 Joint Undertaking (JU) under grant agreement No 806968.
The JU receives support from the European Union's Horizon 2020 research and innovation programme and EFPIA.

1 TABLE OF CONTENT

1 Table of content 1
2 General Information 2
2.1 Contact Details 2
3 ETL Development General 3
3.1 ETL Documentation 3
3.2 ETL Implementation 3
3.3 Record counts data tables 3
3.4 Data density plots 4
3.5 Distinct concepts per person 5
4 Vocabulary Mapping 5
4.1 Vocabularies 5
4.2 Table counts 8
4.3 Mapping Completeness 8
4.4 Drug Mappings 10
4.5 Unmapped Codes 10
4.6 Mapped Codes 11
4.7 Source to concept map 13
5 Technical Infrastructure 14
5.1 CDM Source Table 14
5.2 HADES packages 14
5.3 System Information 14
5.4 Vocabulary Query Performance 15
5.5 Achilles Query Performance 15

2 GENERAL INFORMATION

The goal of the inspection report is to provide insight into the completeness, transparency and quality of the performed Extraction Transform, and Load (ETL) process and the readiness of the data source to be onboarded in the data network to participate in research studies.

2.1 Contact Details

Fill in the table below

items	answers
Data Partner	
Database fullname	fdzformat
Database acronym	HDL examlple data 1
Contact Person	Melissa Finster
Email	Melissa.finster@mevis.fraunhofer.de

3 ETL DEVELOPMENT GENERAL

This section describes the ETL development steps and discusses the quality control steps performed by the SME

3.1 ETL Documentation

https://github.com/FraunhoferMEVIS/ETLfromHDLtoOMOP/tree/main/WhiteRabbit_Documentation

3.2 Record counts data tables

Table 1. Shows the number of records in all clinical data tables

TABLENAME	COUNT	N_PERSONS
cost	48,048,000	NA
observation	35,758,734	6,863,000
visit_occurrence	13,728,000	6,863,000
condition_occurrence	12,422,894	6,806,789
drug_exposure	6,864,000	6,863,000
payer_plan_period	6,864,000	6,863,000
observation_period	6,863,000	6,863,000
person	6,863,000	6,863,000
procedure_occurrence	3,461,788	3,439,024
death	3,431,500	3,431,500
location	17,280	NA
measurement	12,342	6,864
provider	0	NA
specimen	0	0
dose_era	0	0
device_exposure	0	0
visit_detail	0	0
drug_era	0	0
condition_era	0	0
care_site	0	NA
note	0	0

Query executed in 69.55 secs

3.3 Data density plots

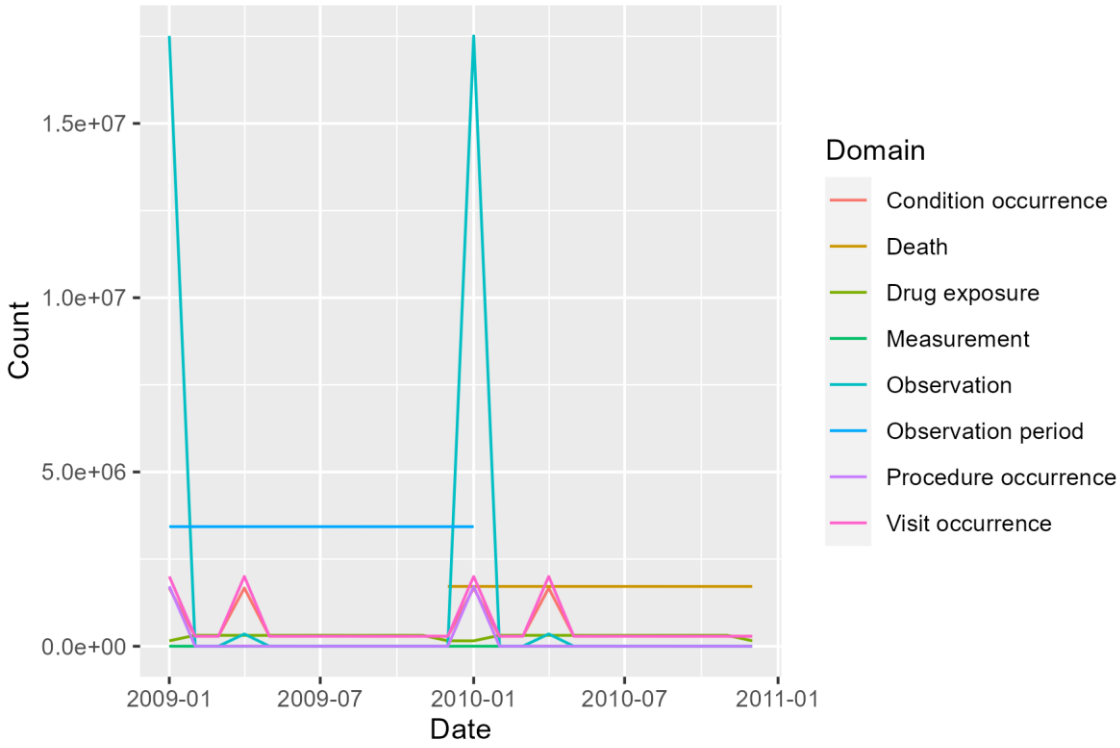

Figure 1. Total record count over time per data domain

3.4 Distinct concepts per person

Table 2. Shows the number of distinct concepts per person for all data domains

Domain	Min	P10	P25	MEDIAN	P75	P90	Max
Observation	1	1	1	1	1	2	4
Drug exposure	1	1	1	1	1	1	1
Condition occurrence	1	1	1	1	1	1	3
Measurement	1	1	1	1	1	1	2

4 Vocabulary Mapping

4.1 Vocabularies

Vocabulary version: v5.0 23-JAN-23

Table 3. The vocabularies available in the CDM with concept count. Note that this does not reflect which concepts are actually used in the clinical CDM tables. S=Standard, C=Classification and '-'=Non-standard

ID	NAME	VERSION	S	C	-
ABMS	Provider Specialty (American Board of Medical Specialties)	2018-06-26 ABMS	85	0	13
ATC	WHO Anatomic Therapeutic Chemical Classification	RxNorm 20210907	0	6,509	231
CDM	OMOP Common DataModel	CDM v6.0.0	1,045	0	0
CMS Place of Service	Place of Service Codes for Professional Claims (CMS)	2009-01-11	51	0	9
Cohort Type	OMOP Cohort Type	NA	0	0	1
Concept Class	OMOP Concept Class	NA	0	0	416
Condition Status	OMOP Condition Status	NA	22	0	0
Condition Type	OMOP Condition Occurrence Type	NA	0	0	118
Cost	OMOP Cost	NA	51	0	0
Cost Type	OMOP Cost Type	NA	0	0	8
CPT4	Current Procedural Terminology version 4 (AMA)	2022 Release	11,909	3,559	1,454
Currency	International Currency Symbol (ISO 4217)	2008	180	0	0
Death Type	OMOP Death Type	NA	0	0	14
Device Type	OMOP Device Type	NA	0	0	4
Domain	OMOP Domain	NA	0	0	65
Drug Type	OMOP Drug Exposure Type	NA	0	0	16
EBM	German Uniform Assessment Standard (EBM)	NA	0	0	3,614
Episode	OMOP Episode	Episode 20201014	14	0	4

ID	NAME	VERSION	S	C	-
Ethnicity	OMOP Ethnicity	NA	2	0	0
Gender	OMOP Gender	NA	2	0	3
HCPCS	Healthcare Common Procedure Coding System (CMS)	20221001 Alpha Numeric HCPCS File	8,461	0	2,808
ICD10CM	International Classification of Diseases, Tenth Revision, Clinical Modification (NCHS)	ICD10CM FY2023 code descriptions	0	0	98,583
ICD10GM	International Classification of Diseases, Tenth Revision, German Edition	ICD10GM 2022	0	0	17,213
ICD9CM	International Classification of Diseases, Ninth Revision, Clinical Modification, Volume 1 and 2 (NCHS)	ICD9CM v32 master descriptions	0	0	17,564
ICD9Proc	International Classification of Diseases, Ninth Revision, Clinical Modification, Volume 3 (NCHS)	ICD9CM v32 master descriptions	2,223	0	2,434
Insured days	Forschungsdatenzentrum (DE) Insured days	2023-03-21	0	0	6
KGV-SV Fachgruppen	Professional groups Insured days	2023-05-03	0	0	73
Korean Revenue Code	Korean Revenue Code (KNHIS)	NA	7	0	0
Language	OMOP Language	Language 20221030	1	0	0
LOINC	Logical Observation Identifiers Names and Codes (Regenstrief Institute)	LOINC 2.73	113,893	49,168	102,015
Meas Type	OMOP Measurement Type	NA	0	0	12
Medicare Specialty	Medicare provider/supplier specialty codes (CMS)	2018-06-26 Specialty	112	0	8
Metadata	OMOP Metadata	NA	1	0	1
NDC	National Drug Code (FDA and manufacturers)	NDC 20230122	11,403	0	1,126,729
None	OMOP Standardized Vocabularies	v5.0 23-JAN-23	0	0	1
Note Type	OMOP Note Type	NA	0	0	10
NUCC	National Uniform Claim Committee Health Care Provider Taxonomy Code Set (NUCC)	2018-06-26 NUCC	674	0	181
Observation Type	OMOP Observation Type	NA	0	0	29
Obs Period Type	OMOP Observation Period Type	NA	0	0	6

ID	NAME	VERSION	S	C	-
OMOP Extension	OMOP Extension (OHDSI)	OMOP Extension 20230110	1,187	0	53
OPS	Operations and Procedures Classification (OPS)	OPS Version 2022	0	0	42,959
OSM	OpenStreetMap (OSMF)	OSM Release 2019-02-21	203,339	0	0
Plan	OMOP Health Plan	NA	11	0	0
Plan Stop Reason	OMOP Plan Stop Reason	NA	13	0	0
Procedure Type	OMOP Procedure Occurrence Type	NA	0	0	97
Race	Race and Ethnicity Code Set (USBC)	Version 1.0	50	0	3
Relationship	OMOP Relationship	NA	14	0	698
Revenue Code	UB04/CMS1450 Revenue Codes (CMS)	2010 Release	538	0	0
RxNorm	RxNorm (NLM)	RxNorm 20230103	149,993	35,341	119,532
RxNorm Extension	OMOP RxNorm Extension	RxNorm Extension 2023-01-16	1,834,465	0	275,964
SNOMED	Systematic Nomenclature of Medicine - Clinical Terms (IHTSDO)	2021-07-31 SNOMED CT International Edition; 2021-09-01 SNOMED CT US Edition; 2021-11-24 SNOMED CT UK Edition	538,283	0	516,652
SOPT	Source of Payment Typology (PHDSC)	SOPT Version 9.2	162	0	6
SPL	Structured Product Labeling (FDA)	NDC 20230122	0	626,507	14,948
Sponsor	OMOP Sponsor	NA	6	0	0
Type Concept	OMOP Type Concept	Type Concept 20221030	80	0	0
UB04 Point of Origin	UB04 Claim Source Inpatient Admission Code (CMS)	NA	0	0	23
UB04 Pri Typ of Adm	UB04 Claim Inpatient Admission Type Code (CMS)	NA	6	0	0
UB04 Pt dis status	UB04 Patient Discharge Status Code (CMS)	NA	0	0	55
UB04 Typ bill	UB04 Type of Bill - Institutional (USHIK)	NA	4	0	294
UCUM	Unified Code for Units of Measure (Regenstrief Institute)	Version 1.8.2	1,029	0	89

ID	NAME	VERSION	S	C	-
US Census	Census regions of the United States (USCB)	US Census 2017 Release	13	0	0
Visit	OMOP Visit	Visit 20211216	19	0	0
Visit Type	OMOP Visit Type	NA	0	0	18
Vocabulary	OMOP Vocabulary	NA	0	0	143

Query executed in 5.44 secs

4.2 Table counts

Table 4. Shows the number of records in all vocabulary tables

TABLENAME	COUNT
concept_class	418
relationship	690
concept	5,945,608
concept_synonym	2,101,511
vocabulary	64
domain	50
concept_ancestor	70,537,715
drug_strength	2,936,738
concept_relationship	46,843,676

Query executed in 23.31 secs

4.3 Mapping Completeness

Table 5. Shows the percentage of codes that are mapped to the standardized vocabularies as well as the percentage of records.

Domain	#Codes Source	#Codes Mapped	%Codes Mapped	#Records Source	#Records Mapped	%Records Mapped
Condition	34,622	6,334	18.3%	12,422,894	2,421,782	19.5%
Condition status	6	5	83.3%	12,422,894	11,042,042	88.9%
Death cause	0	NA	NA	NA	NA	NA
Device	0	NA	NA	NA	NA	NA
Drug	3,432,000	0	0%	6,864,000	0	0%
Measurement	35	35	100%	12,342	12,342	100%
Measurement unit	0	NA	NA	NA	NA	NA
Measurement value	0	NA	NA	NA	NA	NA
Observation	28,665	28,664	100.0%	35,758,734	1,438,734	4.0%
Observation unit	0	NA	NA	NA	NA	NA
Observation value	0	NA	NA	NA	NA	NA
Procedure	79	79	100%	3,461,788	3,461,788	100%

Domain	#Codes Source	#Codes Mapped	%Codes Mapped	#Records Source	#Records Mapped	%Records Mapped
Provider Specialty	0	NA	NA	NA	NA	NA
Specimen	0	NA	NA	NA	NA	NA
Visit	3	2	66.7%	13,728,000	10,296,000	75.0%

Query executed in 41.75 secs

4.4 Drug Mappings

Table 6. The level of the drug mappings

Class	#Records	#Patients	#Codes

Query executed in 2.25 secs

4.5 Unmapped Codes

Table 7 omitted because no unmapped drugs were found.

Query executed in 42.40 secs

Table 8. Top 25 of unmapped conditions. Counts are rounded up to the nearest hundred.

#	Source Value	#Records	#Subjects
1	A550	600	300
2	X9204	600	300
3	U2309	600	300
4	F7204	600	300
5	Q8507	600	300
6	T3707	600	300
7	Q090	600	300
8	G342	600	300
9	X9202	600	300
10	J1307	600	300
11	Q4602	600	300
12	R122	600	300
13	Y110	600	300
14	B3107	600	300
15	O840	600	300
16	H5909	600	300
17	W765	600	300
18	P5202	600	300
19	Y9909	600	300
20	Q1309	600	300
21	I075	600	300
22	S9904	600	300
23	P3309	600	300
24	U235	600	300
25	V546	600	300

Query executed in 32.39 secs

Table 9 omitted because no unmapped measurements were found.

Query executed in 0.10 secs

Table 10. All 1 unmapped observations. Counts are rounded up to the nearest hundred.

#	Source Value	#Records	#Subjects
1	NA	34,320,000	6,863,000

Query executed in 47.83 secs

Table 11 omitted because no unmapped procedures were found.

Query executed in 0.13 secs

Table 12 omitted because no unmapped devices were found.

Query executed in 0.11 secs

Table 13. All 1 unmapped visits. Counts are rounded up to the nearest hundred.

#	Source Value	#Records	#Subjects
1	0	3,432,000	3,431,500

Query executed in 6.11 secs

4.6 Mapped Codes

Table 14 omitted because no mapped drugs were found.

Query executed in 1.51 secs

Table 15. Top 25 of mapped conditions. Counts are rounded up to the nearest hundred.

#	Concept Name	#Records	#Subjects
1	Finding related to pregnancy	45,200	25,000
2	Disorder of upper arm	8,300	4,500
3	Poisoning by drug AND/OR medicinal substance	6,500	3,600
4	Infection due to resistant bacteria	5,800	3,300
5	Abnormal finding on evaluation procedure	5,400	3,000
6	Dislocations/sprains/strains	5,200	2,900
7	Fetal condition affecting obstetrical care of mother	5,200	2,900
8	Cerebrovascular disease	4,800	2,600
9	Pregnancy with abortive outcome	4,200	2,400
10	Muscle and tendon injury	4,100	2,200
11	Disorder of joint of ankle and/or foot	3,800	2,200
12	Acquired absence of organ	3,800	2,200
13	Arthropathy associated with another disorder	3,700	2,100
14	Disorder of lower leg	3,700	2,100
15	Staphylococcal arthritis	3,500	1,900
16	Disorder of sacrum	3,500	2,000
17	Juvenile osteochondrosis of spine	3,500	1,900
18	Disorder of free lower limb	3,500	1,900
19	Gouty arthropathy	3,400	1,900
20	Disorder of forearm	3,400	1,900
21	Purulent myositis	3,300	1,900
22	Postmeningococcal arthritis	3,300	1,800
23	Arthropathy	3,300	1,900
24	Gonococcal bursitis	3,300	1,800
25	Disorder of lumbar spine	3,300	1,800

Query executed in 10.63 secs

Table 16. Top 25 of mapped measurements. Counts are rounded up to the nearest hundred.

#	Concept Name	#Records	#Subjects
1	Screening for disorder	1,300	800
2	HIV CDC stage finding	1,300	800
3	Barthel index	1,000	600
4	Antenatal screening	1,000	600
5	Measurement of Severe acute respiratory syndrome coronavirus 2 (SARS-CoV-2)	600	300
6	Screening for cancer	500	300
7	Albumin measurement	500	300
8	Hematology screening test	500	300
9	Screening for malignant neoplasm of prostate	500	300
10	Alpha-1-Fetoprotein measurement, amniotic fluid	500	300
11	Antenatal screening for isoimmunization	500	300
12	In vivo test of hypersensitivity	500	300
13	Screening for malignant neoplasm of breast	500	300
14	Screening for malignant neoplasm of stomach	500	300
15	Fetal chromosomal abnormality screening	500	300
16	Antibody titer measurement	400	300
17	Blood pressure taking	400	200
18	Immunology laboratory test	400	200
19	Serum/plasma protein test	400	200
20	Blood test	400	200
21	Enzyme measurement	400	200
22	Antenatal screening for malformation	400	200
23	Screening for malignant neoplasm of intestinal tract	200	100
24	Screening for cardiovascular system disease	200	100
25	Globulin measurement	200	100

Query executed in 0.17 secs

Table 17. Top 25 of mapped observations. Counts are rounded up to the nearest hundred.

#	Concept Name	#Records	#Subjects
1	Disorder excluded	1,372,800	1,372,600
2	History of event	11,400	6,500
3	Follow-up encounter	3,200	1,800
4	Family history of clinical finding	2,100	1,200
5	Allergy to substance	1,800	1,000
6	Requires vaccination	1,800	1,000
7	Aftercare	1,700	1,000
8	Patient encounter procedure	1,300	800
9	On waiting list for organ transplant	1,000	600
10	Body mass index 40+ - severely obese	1,000	600
11	Need for personal care assistance	1,000	600
12	Requires polio vaccination	900	500
13	Tobacco user	900	500
14	Exposure to communicable disease	800	500
15	Family history of cancer	800	500
16	Donor for medical or surgical procedure	800	500
17	Blood donor	600	400
18	Mandibular implant in situ	600	300
19	Eye donor for corneal transplant	600	300
20	Very low level of personal hygiene	600	300

#	Concept Name	#Records	#Subjects
21	Accident due to cold of man-made origin	600	300
22	Allergy to drug	600	300
23	Antenatal care of elderly primigravida	600	300
24	Cardiac rehabilitation	600	300
25	Death from direct obstetric cause occurring more than 42 days but less than one year after delivery	600	300

Query executed in 7.15 secs

Table 18. Top 25 of mapped procedures. Counts are rounded up to the nearest hundred.

#	Concept Name	#Records	#Subjects
1	Dialysis procedure	3,432,200	3,431,600
2	Fitting procedure	1,900	1,000
3	Preventive procedure	1,800	1,000
4	Adjustment of implant	1,700	900
5	Attention to device, anastomosis AND/OR stoma	1,600	900
6	Surgical procedure	1,300	800
7	Medical examination for suspected condition	1,300	800
8	Analysis of electronic device used in cardiovascular system	1,000	600
9	Reproductive care management	900	600
10	General examination of patient	900	500
11	Specialized medical examination	800	500
12	Contraception care management	800	500
13	Fitting of prosthesis	700	400
14	Postnatal maternal examination	700	400
15	Child examination	600	300
16	Ophthalmic examination and evaluation	600	300
17	Genetic counseling	600	300
18	Hearing examination	600	300
19	Reversal of sterilization	600	300
20	Examination for suspected mental disorder	600	300
21	Examination for suspected tuberculosis	600	300
22	Pretransplant evaluation of donor	500	300
23	Examination for suspected cardiovascular disease	500	300
24	Examination for injuries	500	300
25	Prophylactic immunotherapy	500	300

Query executed in 6.44 secs

Table 19 omitted because no mapped devices were found.

Query executed in 0.11 secs

Table 20. All 2 mapped visits. Counts are rounded up to the nearest hundred.

#	Concept Name	#Records	#Subjects
1	Outpatient Hospital	6,864,000	6,863,000
2	Outpatient Visit	3,432,000	3,431,500

Query executed in 20.11 secs

4.7 Source to concept map

Note that the full source_to_concept_map table is added in the results.zip

Table 21. Source to concept map breakdown

SOURCE_VOCABULARY_ID	TARGET_VOCABULARY_ID	COUNT

Query executed in 0.17 secs

Note that the full source_to_concept_map table is added in the results.zip

5 Technical Infrastructure

5.1 CDM Source Table

Table 22. cdm_source table content

field	
CDM_SOURCE_NAME	HDL_format1
CDM_SOURCE_ABBREVIATION	MEVIS_format1
CDM_HOLDER	MEVIS
SOURCE_DESCRIPTION	HDL_format1
SOURCE_DOCUMENTATION_REFERENCE	https://github.com/FDZ-Gesundheit/datensatzbeschreibung_fdz_gesundheit
CDM_ETL_REFERENCE	https://github.com/FraunhoferMEVIS/ETLfromHDLtoOMOP/
SOURCE_RELEASE_DATE	2024-06-04
CDM_RELEASE_DATE	2024-09-13
CDM_VERSION	v5.4
CDM_VERSION_CONCEPT_ID	756265
VOCABULARY_VERSION	v5.0 23-JAN-23

5.2 HADES packages

Table 23. Versions of all installed HADES R packages

Package	Version
DatabaseConnector	6.1.0
ParallelLogger	3.3.0
ROhdsiWebApi	1.3.3
SqlRender	1.15.0

Missing HADES packages: CohortMethod, SelfControlledCaseSeries, SelfControlledCohort, EvidenceSynthesis, PatientLevelPrediction, EnsemblePatientLevelPrediction, Capr, CirceR, CohortGenerator, PhenotypeLibrary, EmpiricalCalibration, MethodEvaluation, CohortDiagnostics, Andromeda, BigKnn, Cyclops, Eunomia, FeatureExtraction, Hydra, OhdsiSharing

5.3 System Information

Installed R version: R version 4.2.3 (2023-03-15 ucrt)

System CPU vendor: GenuineIntel

System CPU model: 11th Gen Intel(R) Core(TM) i7-1165G7 @ 2.80GHz

System CPU number of cores: 8

System RAM: 34.07 GB

DBMS: postgresql

WebAPI version: unknown

5.4 Vocabulary Query Performance

The number of 'Maps To' relations is equal to 4372361. This query was executed in 19.96 secs

5.5 Achilles Query Performance

Table 24. Execution time of queries of the Achilles R-Package

ID	NAME	DURATION
0	Source name	7.57
1	Number of persons	2.98
2	Number of persons by gender	4.47
3	Number of persons by year of birth	7.92
4	Number of persons by race	8.08
5	Number of persons by ethnicity	7.68
7	Number of persons with invalid provider_id	2.1
8	Number of persons with invalid location_id	1.04
9	Number of persons with invalid care_site_id	0.59
10	Number of all persons by year of birth by gender	7.51
11	Number of non-deceased persons by year of birth by gender	9.34
12	Number of persons by race and ethnicity	8.56
101	Number of persons by age, with age at first observation period	7.42
102	Number of persons by gender by age, with age at first observation period	6.6
108	Number of persons by length of observation period, in 30d increments	30.39
109	Number of persons with continuous observation in each year	50.96
110	Number of persons with continuous observation in each month	39.04
111	Number of persons by observation period start month	15.1
112	Number of persons by observation period end month	16.95
113	Number of persons by number of observation periods	10.97
114	Number of persons with observation period before year-of-birth	9.66
115	Number of persons with observation period end < observation period start	0.44
116	Number of persons with at least one day of observation in each year by gender and age decile	47.46
118	Number of observation periods with invalid person_id	8.32
119	Number of observation period records by period_type_concept_id	1.06
200	Number of persons with at least one visit occurrence, by visit_concept_id	25.82
201	Number of visit occurrence records, by visit_concept_id	12.44
202	Number of persons by visit occurrence start month, by visit_concept_id	33.6
204	Number of persons with at least one visit occurrence, by visit_concept_id by calendar year by gender by age decile	59.59
207	Number of visit records with invalid person_id	9.05
209	Number of visit records with invalid care_site_id	0.95
210	Number of visit_occurrence records outside a valid observation period	10.65
211	Number of visit records with end date < start date	0.87

ID	NAME	DURATION
212	Number of persons with at least one visit occurrence, by calendar year by gender by age decile	46.08
220	Number of visit occurrence records by visit occurrence start month	15.32
221	Number of persons by visit start year	28.48
225	Number of visit_occurrence records by visit_source_concept_id	11.77
230	Number of visit_occurrence records inside valid observation period	11.48
231	Proportion of people with at least one visit_occurrence record outside a valid observation period	19.91
232	Proportion of visit_occurrence records outside a valid observation period	10.3
300	Number of providers	0.03
301	Number of providers by specialty concept_id	0.02
303	Number of providers records by specialty_concept_id and visit_concept_id	0.14
325	Number of provider records by specialty_source_concept_id	0.04
400	Number of persons with at least one condition occurrence, by condition_concept_id	23.5
401	Number of condition occurrence records, by condition_concept_id	12.04
402	Number of persons by condition occurrence start month, by condition_concept_id	30.73
404	Number of persons with at least one condition occurrence, by condition_concept_id by calendar year by gender by age decile	54.53
405	Number of condition occurrence records, by condition_concept_id by condition_type_concept_id	12.43
409	Number of condition occurrence records with invalid person_id	9.5
410	Number of condition occurrence records outside valid observation period	10.43
411	Number of condition occurrence records with end date < start date	0.83
412	Number of condition occurrence records with invalid provider_id	0.87
413	Number of condition occurrence records with invalid visit_id	17.08
414	Number of condition occurrence records, by condition_status_concept_id	11.62
415	Number of condition occurrence records, by condition_type_concept_id	11.27
416	Number of condition occurrence records, by condition_status_concept_id, condition_type_concept_id	11.73
420	Number of condition occurrence records by condition occurrence start month	14.27
425	Number of condition_occurrence records by condition_source_concept_id	11.8
424	Number of distinct people with co-occurring condition_occurrence condition_concept_id pairs	4.98
430	Number of condition_occurrence records inside a valid observation period	10.78
431	Proportion of people with at least one condition_occurrence record outside a valid observation period	18.2
432	Proportion of condition_occurrence records outside a valid observation period	10.31
500	Number of persons with death, by cause_concept_id	8.36
501	Number of records of death, by cause_concept_id	5.72
502	Number of persons by death month	10.17
504	Number of persons with a death, by calendar year by gender by age decile	22.84
505	Number of death records, by death_type_concept_id	5.59
509	Number of death records with invalid person_id	7.57
510	Number of death records outside valid observation period	7.93
525	Number of death records by cause_source_concept_id	0.53
530	Number of death records inside a valid observation period	5.13

ID	NAME	DURATION
531	Proportion of people with at least one death record outside a valid observation period	8.79
532	Proportion of death records that occur outside a valid observation period	7.59
600	Number of persons with at least one procedure occurrence, by procedure_concept_id	8.62
601	Number of procedure occurrence records, by procedure_concept_id	5.82
602	Number of persons by procedure occurrence start month, by procedure_concept_id	10.56
604	Number of persons with at least one procedure occurrence, by procedure_concept_id by calendar year by gender by age decile	22.29
605	Number of procedure occurrence records, by procedure_concept_id by procedure_type_concept_id	5.94
609	Number of procedure occurrence records with invalid person_id	7.56
610	Number of procedure occurrence records outside valid observation period	7.91
612	Number of procedure occurrence records with invalid provider_id	0.3
613	Number of procedure occurrence records with invalid visit_id	2.49
620	Number of procedure occurrence records by procedure occurrence start month	6.57
625	Number of procedure_occurrence records by procedure_source_concept_id	5.93
624	Number of distinct people with co-occurring procedure_occurrence procedure_concept_id pairs	4.25
630	Number of procedure_occurrence records inside a valid observation period	5.32
631	Proportion of people with at least one procedure_occurrence record outside a valid observation period	9.36
632	Proportion of procedure_occurrence records outside a valid observation period	8.09
691	Percentage of total persons that have at least x procedures	9.71
700	Number of persons with at least one drug exposure, by drug_concept_id	15.2
701	Number of drug exposure records, by drug_concept_id	9.52
702	Number of persons by drug exposure start month, by drug_concept_id	20.08
704	Number of persons with at least one drug exposure, by drug_concept_id by calendar year by gender by age decile	35.84
705	Number of drug exposure records, by drug_concept_id by drug_type_concept_id	9.97
709	Number of drug exposure records with invalid person_id	8.46
710	Number of drug exposure records outside valid observation period	8.95
711	Number of drug exposure records with end date < start date	0.59
712	Number of drug exposure records with invalid provider_id	0.64
713	Number of drug exposure records with invalid visit_id	0.03
720	Number of drug exposure records by drug exposure start month	11.31
725	Number of drug_exposure records by drug_source_concept_id	9.55
724	Number of distinct people with co-occurring drug_exposure drug_concept_id pairs	0.49
730	Number of drug_exposure records inside a valid observation period	8.95
731	Proportion of people with at least one drug_exposure record outside a valid observation period	11.96
732	Proportion of drug_exposure records outside a valid observation period	9.23
791	Percentage of total persons that have at least x drug exposures	18.12
800	Number of persons with at least one observation occurrence, by observation_concept_id	51.6

ID	NAME	DURATION
801	Number of observation occurrence records, by observation_concept_id	18.18
802	Number of persons by observation occurrence start month, by observation_concept_id	1.18
804	Number of persons with at least one observation occurrence, by observation_concept_id by calendar year by gender by age decile	2.23
805	Number of observation occurrence records, by observation_concept_id by observation_type_concept_id	18.31
807	Number of observation occurrence records, by observation_concept_id and unit_concept_id	19.12
809	Number of observation records with invalid person_id	13.74
810	Number of observation records outside valid observation period	15.15
812	Number of observation records with invalid provider_id	2.37
813	Number of observation records with invalid visit_id	17.21
814	Number of observation records with no value (numeric, string, or concept)	2.29
820	Number of observation records by observation start month	26.75
822	Number of observation records, by observation_concept_id and value_as_concept_id	18.37
823	Number of observation records, by observation_concept_id and qualifier_concept_id	18.47
824	Number of distinct people with co-occurring observation observation_concept_id pairs	3.65
825	Number of observation records by observation_source_concept_id	18.33
826	Number of observation records by value_as_concept_id	17.53
827	Number of observation records by unit_concept_id	17.54
830	Number of observation records inside a valid observation period	16.14
831	Proportion of people with at least one observation record outside a valid observation period	43.2
832	Proportion of observation records outside a valid observation period	15.68
891	Percentage of total persons that have at least x observations	34.91
900	Number of persons with at least one drug era, by drug_concept_id	0.07
901	Number of drug era records, by drug_concept_id	0.03
902	Number of persons by drug era start month, by drug_concept_id	0.03
904	Number of persons with at least one drug era, by drug_concept_id by calendar year by gender by age decile	0.04
908	Number of drug eras without valid person	0.03
910	Number of drug_era records outside valid observation period	0.03
911	Number of drug eras with end date < start date	0.03
920	Number of drug era records by drug era start month	0.03
930	Number of drug_era records inside a valid observation period	0.03
931	Proportion of people with at least one drug_era record outside a valid observation period	0.03
932	Proportion of drug_era records outside a valid observation period	0.04
1,000	Number of persons with at least one condition era, by condition_concept_id	0.07
1,001	Number of condition era records, by condition_concept_id	0.04
1,002	Number of persons by condition era start month, by condition_concept_id	0.03
1,004	Number of persons with at least one condition era, by condition_concept_id by calendar year by gender by age decile	0.03
1,008	Number of condition eras without valid person	0.04
1,010	Number of condition_era records outside a valid observation period	0.03
1,011	Number of condition eras with end date < start date	0.03

ID	NAME	DURATION
1,020	Number of condition era records by condition era start month	0.03
1,030	Number of condition_era records inside a valid observation period	0.03
1,031	Proportion of people with at least one condition_era record outside a valid observation period	0.13
1,032	Proportion of condition_era records outside a valid observation period	0.04
1,100	Number of persons by location 3-digit zip	12.72
1,101	Number of persons by location state	12.21
1,102	Number of care sites by location 3-digit zip	0.03
1,103	Number of care sites by location state	0.03
1,200	Number of persons by place of service	0.6
1,201	Number of visits by place of service	0.87
1,202	Number of care sites by place of service	0.03
1,203	Number of visits by place of service discharge type	1.1
1,300	Number of persons with at least one visit detail, by visit_detail_concept_id	0.07
1,301	Number of visit detail records, by visit_detail_concept_id	0.03
1,302	Number of persons by visit detail start month, by visit_detail_concept_id	0.03
1,304	Number of persons with at least one visit detail, by visit_detail_concept_id by calendar year by gender by age decile	0.03
1,307	Number of visit records with invalid person_id	0.05
1,309	Number of visit_detail records with invalid care_site_id	0.03
1,310	Number of visit_detail records outside a valid observation period	0.03
1,311	Number of visit_detail records with end date < start date	0.03
1,312	Number of persons with at least one visit detail, by calendar year by gender by age decile	0.03
1,320	Number of visit detail records by visit detail start month	0.03
1,321	Number of persons by visit start year	0.03
1,325	Number of visit_detail records by visit_detail_source_concept_id	0.03
1,326	Number of records by domain by visit_detail_concept_id	11.07
1,330	Number of visit_detail records inside a valid observation period	0.06
1,331	Proportion of people with at least one visit_detail record outside a valid observation period	0.03
1,332	Proportion of visit_detail records outside a valid observation period	0.03
1,408	Number of persons by length of payer plan period, in 30d increments	31.4
1,409	Number of persons with continuous payer plan in each year	28.93
1,410	Number of persons with continuous payer plan in each month	49.22
1,411	Number of persons by payer plan period start month	19.14
1,412	Number of persons by payer plan period end month	19.54
1,413	Number of persons by number of payer plan periods	13.74
1,414	Number of persons with payer plan period before year-of-birth	9.29
1,415	Number of persons with payer plan period end < payer plan period start	0.43
1,425	Number of payer_plan_period records by payer_source_concept_id	0.85
1,610	Number of records by revenue_code_concept_id	2.34
1,800	Number of persons with at least one measurement occurrence, by measurement_concept_id	0.48
1,801	Number of measurement occurrence records, by measurement_concept_id	0.1
1,802	Number of persons by measurement occurrence start month, by measurement_concept_id	0.12
1,804	Number of persons with at least one mesurement occurrence, by measurement_concept_id by calendar year by gender by age decile	0.27

ID	NAME	DURATION
1,805	Number of measurement occurrence records, by measurement_concept_id by measurement_type_concept_id	0.11
1,807	Number of measurement occurrence records, by measurement_concept_id and unit_concept_id	0.11
1,809	Number of measurement records with invalid person_id	0.13
1,810	Number of measurement records outside valid observation period	0.13
1,811	Number of measurement records with a valid value (with a mapped, non-null value_as_number)	0.03
1,812	Number of measurement records with invalid provider_id	0.03
1,813	Number of measurement records with invalid visit_id	0.34
1,814	Number of measurement records with no value (numeric, string, or concept)	0.16
1,818	Number of measurement records below/within/above normal range, by measurement_concept_id and unit_concept_id	0.07
1,819	Number of measurement records, by measurement_concept_id, with a valid value (with a mapped, non-null value_as_number)	0.03
1,820	Number of measurement records by measurement start month	0.15
1,821	Number of measurement records with no numeric value	0.04
1,822	Number of measurement records, by measurement_concept_id and value_as_concept_id	0.13
1,823	Number of measurement records, by measurement_concept_id and operator_concept_id	0.1
1,824	Number of distinct people with co-occurring measurement measurement_concept_id pairs	0.05
1,825	Number of measurement records by measurement_source_concept_id	0.1
1,826	Number of measurement records by value_as_concept_id	0.11
1,827	Number of measurement records by unit_concept_id	0.11
1,830	Number of visit_detail records inside a valid observation period	0.09
1,831	Proportion of people with at least one measurement record outside a valid observation period	0.08
1,832	Proportion of measurement records outside a valid observation period	0.12
1,833	Proportion of measurement records inside a valid observation period and without a value	0.16
1,891	Percentage of total persons that have at least x measurements	0.11
1,900	Source values mapped to concept_id 0 by table, by column, by source_value	1.44
2,003	Number of patients with at least 1 Visit	20.18
2,004	Number of distinct patients that overlap between specific domains	21.28
2,100	Number of persons with at least one device exposure, by device_concept_id	0.09
2,101	Number of device exposure records, by device_concept_id	0.03
2,102	Number of persons by device records start month, by device_concept_id	0.05
2,104	Number of persons with at least one device exposure, by device_concept_id by calendar year by gender by age decile	0.07
2,105	Number of device exposure records, by device_concept_id by device_type_concept_id	0.03
2,110	Number of device_exposure records outside valid observation period	0.04
2,125	Number of device_exposure records by device_source_concept_id	0.04
2,130	Number of device_exposure records inside a valid observation period	0.15
2,131	Proportion of people with at least one device_exposure record outside a valid observation period	0.06
2,132	Proportion of device_exposure records outside a valid observation period	0.07
2,191	Percentage of total persons that have at least x device exposures	0.03

ID	NAME	DURATION
2,200	Number of persons with at least one note by note_type_concept_id	0.08
2,201	Number of note records, by note_type_concept_id	0.03

Query executed in 0.25 secs
