# Supplementary material for: ETL: From the German Health Data Lab data formats to the OMOP Common Data Model
Source: PLoS One. 2025 Jan 6;20(1):e0311511. doi: 10.1371/journal.pone.0311511 (PMC11703056; doi:10.1371/journal.pone.0311511)
Supplement: S2 File — (PDF) [file pone.0311511.s004.pdf]

CDM Inspection report for the fdzformatnew database

Package Version: 1.2.4

Date: Mon Sep 2 16:20:48 2024

Authors: mfinster

This project has received funding from the Innovative Medicines Initiative 2 Joint Undertaking (JU) under grant agreement No 806968.
The JU receives support from the European Union's Horizon 2020 research and innovation programme and EFPIA.

1 TABLE OF CONTENT

1 Table of content 1
2 General Information 2
2.1 Contact Details 2
3 ETL Development General 3
3.1 ETL Documentation 3
3.2 Record counts data tables 3
3.3 Data density plots 4
3.4 Distinct concepts per person 5
4 Vocabulary Mapping 5
4.1 Vocabularies 5
4.2 Table counts 8
4.3 Mapping Completeness 8
4.4 Drug Mappings 10
4.5 Unmapped Codes 10
4.6 Mapped Codes 12
4.7 Source to concept map 15
5 Technical Infrastructure 15
5.1 CDM Source Table 15
5.2 HADES packages 15
5.3 System Information 16
5.4 Vocabulary Query Performance 16
5.5 Achilles Query Performance 16

2 GENERAL INFORMATION

The goal of the inspection report is to provide insight into the completeness, transparency and quality of the performed Extraction Transform, and Load (ETL) process and the readiness of the data source to be onboarded in the data network to participate in research studies.

2.1 Contact Details

Items	answers
Data Partner	
Database fullname	fdzformat
Database acronym	HDL examlple data 3
Contact Person	Melissa Finster
Email	Melissa.finster@mevis.fraunhofer.de

3 ETL DEVELOPMENT GENERAL

This section describes the ETL development steps and discusses the quality control steps performed by the SME

3.1 ETL Documentation

https://github.com/FraunhoferMEVIS/ETLfromHDLtoOMOP/tree/main/WhiteRabbit_Documentation

3.2 Record counts data tables

Table 1. Shows the number of records in all clinical data tables

TABLENAME	COUNT	N_PERSONS
procedure_occurrence	9,922,480	100,000
Observation	8,052,039	100,000
condition_occurrence	4,985,719	100,000
Cost	3,724,642	NA
visit_occurrence	1,624,715	100,000
drug_exposure	1,604,096	100,000
Provider	1,567,763	NA
care_site	801,756	NA
Person	100,001	100,001
observation_period	100,000	100,000
payer_plan_period	100,000	100,000
Location	8,228	NA
Measurement	4,509	3,120
Death	1,221	1,221
Specimen	0	0
dose_era	0	0
device_exposure	0	0
visit_detail	0	0
drug_era	0	0
condition_era	0	0
Note	0	0

Query executed in 18.77 secs

3.3 Data density plots

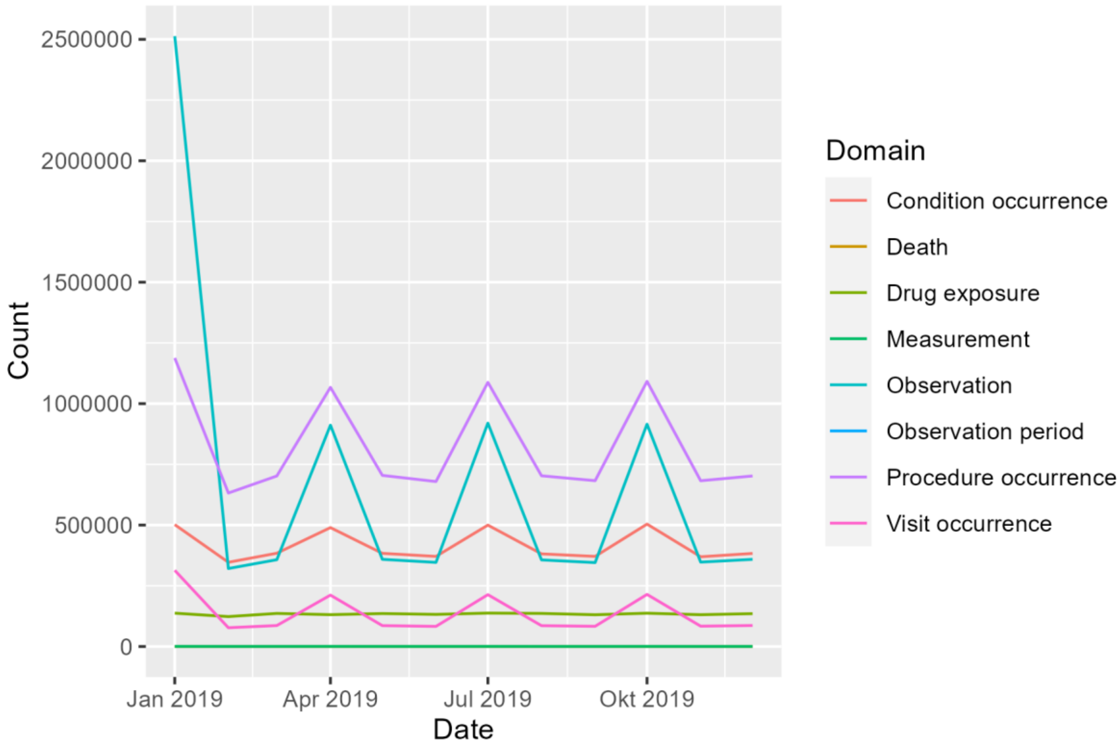

Figure 1. Total record count over time per data domain

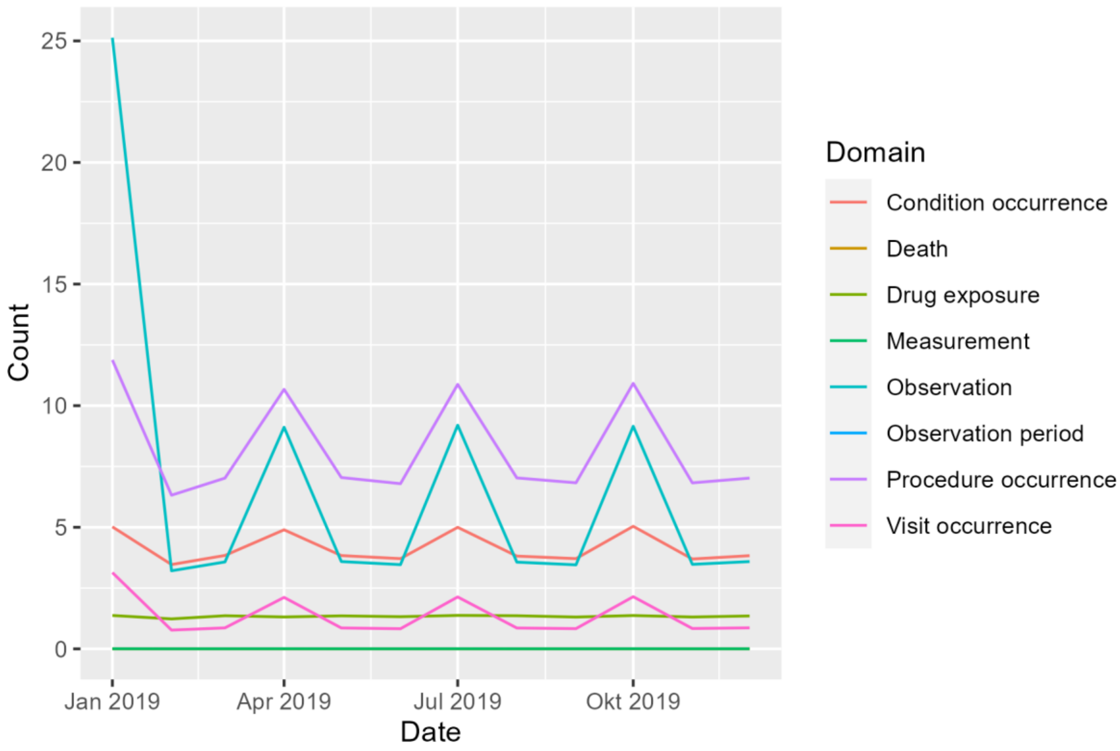

Figure 2. Number of records per person over time per data domain

3.4 Distinct concepts per person

Table 2. Shows the number of distinct concepts per person for all data domains

Domain	Min	P10	P25	MEDIAN	P75	P90	Max
Measurement	1	1	1	1	1	1	3
Condition occurrence	16	25	26	28	31	33	43
Procedure occurrence	7	13	15	17	19	21	32
Observation	2	2	3	3	4	4	8
Drug exposure	1	1	1	1	1	1	3

4 Vocabulary Mapping

4.1 Vocabularies

Vocabulary version: v5.0 23-JAN-23

Table 3. The vocabularies available in the CDM with concept count. Note that this does not reflect which concepts are actually used in the clinical CDM tables. S=Standard, C=Classification and '-'=Non-standard

ID	NAME	VERSION	S	C	-
ABMS	Provider Specialty (American Board of Medical Specialties)	2018-06-26 ABMS	85	0	13
ATC	WHO Anatomic Therapeutic Chemical Classification	RxNorm 20210907	0	6,509	231
CDM	OMOP Common DataModel	CDM v6.0.0	1,045	0	0
CMS Place of Service	Place of Service Codes for Professional Claims (CMS)	2009-01-11	51	0	9
Cohort Type	OMOP Cohort Type	NA	0	0	1
Concept Class	OMOP Concept Class	NA	0	0	416
Condition Status	OMOP Condition Status	NA	22	0	0
Condition Type	OMOP Condition Occurrence Type	NA	0	0	118
Cost	OMOP Cost	NA	51	0	0
Cost Type	OMOP Cost Type	NA	0	0	8
CPT4	Current Procedural Terminology version 4 (AMA)	2022 Release	11,909	3,559	1,454
Currency	International Currency Symbol (ISO 4217)	2008	180	0	0
Death Type	OMOP Death Type	NA	0	0	14
Device Type	OMOP Device Type	NA	0	0	4
Domain	OMOP Domain	NA	0	0	65
Drug Type	OMOP Drug Exposure Type	NA	0	0	16
EBM	German Uniform Assessment Standard (EBM)	NA	0	0	3,614

ID	NAME	VERSION	S	C	-
Episode	OMOP Episode	Episode 20201014	14	0	4
Ethnicity	OMOP Ethnicity	NA	2	0	0
Gender	OMOP Gender	NA	2	0	3
HCPCS	Healthcare Common Procedure Coding System (CMS)	20221001 Alpha Numeric HCPCS File	8,461	0	2,808
ICD10CM	International Classification of Diseases, Tenth Revision, Clinical Modification (NCHS)	ICD10CM FY2023 code descriptions	0	0	98,583
ICD10GM	International Classification of Diseases, Tenth Revision, German Edition	ICD10GM 2022	0	0	17,213
ICD9CM	International Classification of Diseases, Ninth Revision, Clinical Modification, Volume 1 and 2 (NCHS)	ICD9CM v32 master descriptions	0	0	17,564
ICD9Proc	International Classification of Diseases, Ninth Revision, Clinical Modification, Volume 3 (NCHS)	ICD9CM v32 master descriptions	2,223	0	2,434
Insured days	Forschungsdatenzentrum (DE) Insured days	2023-03-21	0	0	6
KGV-SV Fachgruppen	Professional groups Insured days	2023-05-03	0	0	73
Korean Revenue Code	Korean Revenue Code (KNHIS)	NA	7	0	0
Language	OMOP Language	Language 20221030	1	0	0
LOINC	Logical Observation Identifiers Names and Codes (Regenstrief Institute)	LOINC 2.73	113,893	49,168	102,015
Meas Type	OMOP Measurement Type	NA	0	0	12
Medicare Specialty	Medicare provider/supplier specialty codes (CMS)	2018-06-26 Specialty	112	0	8
Metadata	OMOP Metadata	NA	1	0	1
NDC	National Drug Code (FDA and manufacturers)	NDC 20230122	11,403	0	1,126,729
None	OMOP Standardized Vocabularies	v5.0 23-JAN-23	0	0	1
Note Type	OMOP Note Type	NA	0	0	10
NUCC	National Uniform Claim Committee Health Care Provider Taxonomy Code Set (NUCC)	2018-06-26 NUCC	674	0	181
Observation Type	OMOP Observation Type	NA	0	0	29

ID	NAME	VERSION	S	C	-
Obs Period Type	OMOP Observation Period Type	NA	0	0	6
OMOP Extension	OMOP Extension (OHDSI)	OMOP Extension 20230110	1,187	0	53
OPS	Operations and Procedures Classification (OPS)	OPS Version 2022	0	0	42,959
OSM	OpenStreetMap (OSMF)	OSM Release 2019-02-21	203,339	0	0
Plan	OMOP Health Plan	NA	11	0	0
Plan Stop Reason	OMOP Plan Stop Reason	NA	13	0	0
Procedure Type	OMOP Procedure Occurrence Type	NA	0	0	97
Race	Race and Ethnicity Code Set (USBC)	Version 1.0	50	0	3
Relationship	OMOP Relationship	NA	14	0	698
Revenue Code	UB04/CMS1450 Revenue Codes (CMS)	2010 Release	538	0	0
RxNorm	RxNorm (NLM)	RxNorm 20230103	149,993	35,341	119,532
RxNorm Extension	OMOP RxNorm Extension	RxNorm Extension 2023-01-16	1,834,465	0	275,964
SNOMED	Systematic Nomenclature of Medicine - Clinical Terms (IHTSDO)	2021-07-31 SNOMED CT International Edition; 2021-09-01 SNOMED CT US Edition; 2021-11-24 SNOMED CT UK Edition	538,283	0	516,652
SOPT	Source of Payment Typology (PHDSC)	SOPT Version 9.2	162	0	6
SPL	Structured Product Labeling (FDA)	NDC 20230122	0	626,507	14,948
Sponsor	OMOP Sponsor	NA	6	0	0
Type Concept	OMOP Type Concept	Type Concept 20221030	80	0	0
UB04 Point of Origin	UB04 Claim Source Inpatient Admission Code (CMS)	NA	0	0	23
UB04 Pri Typ of Adm	UB04 Claim Inpatient Admission Type Code (CMS)	NA	6	0	0
UB04 Pt dis status	UB04 Patient Discharge Status Code (CMS)	NA	0	0	55
UB04 Typ bill	UB04 Type of Bill - Institutional (USHIK)	NA	4	0	294

ID	NAME	VERSION	S	C	-
UCUM	Unified Code for Units of Measure (Regenstrief Institute)	Version 1.8.2	1,029	0	89
US Census	Census regions of the United States (USCB)	US Census 2017 Release	13	0	0
Visit	OMOP Visit	Visit 20211216	19	0	0
Visit Type	OMOP Visit Type	NA	0	0	18
Vocabulary	OMOP Vocabulary	NA	0	0	143

Query executed in 11.05 secs

4.2 Table counts

Table 4. Shows the number of records in all vocabulary tables

TABLENAME	COUNT
concept_class	418
relationship	690
concept	5,945,608
concept_synonym	2,101,511
vocabulary	64
domain	50
concept_ancestor	70,537,715
drug_strength	2,936,738
concept_relationship	46,843,676

Query executed in 21.47 secs

4.3 Mapping Completeness

Table 5. Shows the percentage of codes that are mapped to the standardized vocabularies as well as the percentage of records.

Domain	#Codes Source	#Codes Mapped	%Codes Mapped	#Records Source	#Records Mapped	%Records Mapped
Condition	53,122	53,003	99.8%	4,985,719	4,979,108	99.9%
Condition status	4	4	100%	4,985,719	4,985,719	100%
Death cause	0	NA	NA	NA	NA	NA
Device	0	NA	NA	NA	NA	NA
Drug	12,726	148	1.2%	1,604,096	2,251	0.1%
Measurement	330	330	100%	4,509	4,509	100%
Measurement unit	0	NA	NA	NA	NA	NA
Measurement value	0	NA	NA	NA	NA	NA
Observation	6,389,435	11,917	0.2%	8,052,039	1,093,191	13.6%
Observation unit	0	NA	NA	NA	NA	NA

Domain	#Codes Source	#Codes Mapped	%Codes Mapped	#Records Source	#Records Mapped	%Records Mapped
Observation value	0	NA	NA	NA	NA	NA
Procedure	1,504,196	32,684	2.2%	9,922,480	3,019,807	30.4%
Provider Specialty	132	101	76.5%	1,567,763	821,794	52.4%
Specimen	0	NA	NA	NA	NA	NA
Visit	173	172	99.4%	1,624,715	1,212,814	74.6%

Query executed in 77.18 secs

4.4 Drug Mappings

Table 6. The level of the drug mappings

Class	#Records	#Patients	#Codes
Ingredient	2,251	1,844	148

Query executed in 1.63 secs

4.5 Unmapped Codes

Table 7. Top 25 of unmapped drugs. Counts are rounded up to the nearest hundred.

#	Source Value	#Records	#Subjects
1	19082360	200	200
2	18347365	200	200
3	03214486	200	200
4	11716109	200	200
5	07361735	200	200
6	03699710	200	200
7	18342244	200	200
8	17228871	200	200
9	03697728	200	200
10	04694040	200	200
11	14753677	200	200
12	04697707	200	200
13	06755389	200	200
14	12858751	200	200
15	11717480	200	200
16	03699590	200	200
17	12860268	200	200
18	04670677	200	200
19	01860207	200	200
20	02023198	200	200
21	09111988	200	200
22	17296080	200	200
23	06845419	200	200
24	05366349	200	200
25	12861546	200	200

Query executed in 6.40 secs

Table 8. Top 25 of unmapped conditions. Counts are rounded up to the nearest hundred.

#	Source Value	#Records	#Subjects
1	M312,	400	300
2	K5588,	300	200
3	K5781,	300	200
4	K589,	300	200
5	K5701,	300	200
6	K5700,	300	200
7	K5780,	300	200
8	K5741,	200	200

#	Source Value	#Records	#Subjects
9	K5581,	200	200
10	K5740,	200	200
11	K580,	200	200
12	K5582,	200	200
13	M312,R	200	200
14	K5720,	200	200
15	K5721,	200	200
16	K5582,R	200	100
17	K5721,R	200	100
18	K5781,R	200	100
19	K580,R	200	100
20	M312,B	200	100
21	K5740,R	200	100
22	K5780,R	100	100
23	K589,R	100	100
24	K5700,R	100	100
25	K5741,R	100	100

Query executed in 0.96 secs

Table 9 omitted because no unmapped measurements were found.

Query executed in 0.15 secs

Table 10. All 1 unmapped observations. Counts are rounded up to the nearest hundred.

#	Source Value	#Records	#Subjects
1	NA	558,900	1e+05

Query executed in 44.80 secs

Table 11. Top 25 of unmapped procedures. Counts are rounded up to the nearest hundred.

#	Source Value	#Records	#Subjects
1	5-810.91,	300	200
2	5-810.30,	200	200
3	5-787.4f,	200	200
4	5-794.79,	200	100
5	5-800.1t,	200	200
6	5-796.60,	200	100
7	5-79b.68,	200	200
8	5-787.9k,	200	200
9	5-795.2b,	200	100
10	5-790.x0,	200	100
11	5-801.m7,	200	200
12	5-801.ck,	200	100
13	5-79b.eb,	200	100
14	5-794.ak,	200	100
15	5-809.16,	200	100
16	5-793.hn,	200	100
17	5-801.cq,	200	100
18	5-794.ex,	200	100
19	5-800.a3,	200	100
20	5-790.0j,	200	100

#	Source Value	#Records	#Subjects
21	5-79a.ch,	200	100
22	5-794.3h,	200	100
23	5-811.xp,	200	200
24	5-819.0e,	200	100
25	5-79b.6j,	200	100

Query executed in 38.41 secs

Table 12 omitted because no unmapped devices were found.

Query executed in 0.14 secs

Table 13. All 1 unmapped visits. Counts are rounded up to the nearest hundred.

#	Source Value	#Records	#Subjects
1	NA	412,000	93,800

Query executed in 0.74 secs

4.6 Mapped Codes

Table 14. All 18 mapped drugs. Counts are rounded up to the nearest hundred.

#	Concept Name	#Records	#Subjects
1	lenograstim	400	300
2	fibrinogen	300	300
3	prothrombin	200	200
4	factor XIII	200	200
5	infliximab	200	200
6	nitric oxide	200	200
7	rituximab	200	200
8	palivizumab	200	200
9	rasburicase	100	100
10	asparaginase	100	100
11	dexrazoxane	100	100
12	pegfilgrastim	100	100
13	thiotepa	100	100
14	nusinersen	100	100
15	letermovir	100	100
16	vedolizumab	100	100
17	ruxolitinib	100	100
18	vipivotide tetraxetan	100	100

Query executed in 0.63 secs

Table 15. Top 25 of mapped conditions. Counts are rounded up to the nearest hundred.

#	Concept Name	#Records	#Subjects
1	Disorder of forearm	104,200	46,700
2	Disorder of upper arm	100,900	45,600
3	Disorder of lower leg	79,800	38,300
4	Finding related to pregnancy	77,600	37,300
5	Disorder of lumbar spine	64,000	32,000
6	Disorder of cervical spine	62,600	31,300
7	Disorder of thoracic spine	60,600	30,700
8	Disorder of sacrum	55,100	28,200

#	Concept Name	#Records	#Subjects
9	Disorder of shoulder	52,200	27,000
10	Disorder of hand	50,800	26,400
11	Disorder of free lower limb	49,600	25,700
12	Disorder of bone	35,500	19,100
13	Disorder of joint of ankle and/or foot	31,600	17,200
14	Disorder of musculoskeletal system	25,600	14,300
15	Arthropathy of multiple joints	25,500	14,100
16	Arthropathy	24,700	13,700
17	Post-infective arthritis	23,400	13,100
18	Disorder of muscle	23,200	13,000
19	Bone necrosis	22,700	12,800
20	Disorder of connective tissue	20,600	11,600
21	Lower limb joint arthritis	20,400	11,500
22	Juvenile rheumatoid arthritis	19,800	11,100
23	Rheumatoid arthritis	19,100	10,800
24	Arthritis of spine	16,900	9,700
25	Infective myositis	16,700	9,500

Query executed in 32.93 secs

Table 16. Top 25 of mapped measurements. Counts are rounded up to the nearest hundred.

#	Concept Name	#Records	#Subjects
1	Histopathology test	1,100	800
2	Serum/plasma protein test	500	300
3	Immunology laboratory test	400	200
4	Histologic test	300	300
5	Enzyme measurement	300	200
6	Blood test	300	200
7	Measurement of level of substance in blood	200	200
8	Hormone measurement	200	100
9	Antibody titer measurement	200	100
10	Red blood cell test	100	100
11	Measurement of respiratory function	100	100
12	Globulin measurement	100	100
13	Alpha-1-Fetoprotein measurement	100	100
14	Albumin measurement	100	100
15	Dynamic endocrine function test	100	100
16	Cerebrospinal fluid pressure recording	100	100
17	Carbon monoxide diffusing capacity measurement	100	100
18	In vivo test of hypersensitivity	100	100
19	6 minute walk test distance	100	100
20	Calibration of urethra	100	100
21	Anorectal manometry	100	100
22	Inhalation bronchial challenge testing	100	100
23	Invasive oxygen saturation monitoring	100	100
24	Spirometry	100	100
25	Esophageal manometry	100	100

Query executed in 0.26 secs

Table 17. Top 25 of mapped observations. Counts are rounded up to the nearest hundred.

#	Concept Name	#Records	#Subjects
1	Childbirth	816,400	100,000
2	Disorder excluded	165,300	56,100
3	Accidental event	84,900	35,800
4	Emergency	7,000	6,900
5	Injury whilst engaged in work activity	2,100	2,000
6	Intensive care monitoring	1,100	1,100
7	Feeding problems in newborn	900	600
8	Maternal death	900	600
9	Death from any obstetric cause occurring more than 42 days but less than one year after delivery	600	400
10	Antenatal care	400	300
11	Excessive weight gain	400	300
12	Palliative care	400	400
13	Suspected fetal damage from viral disease in mother	400	300
14	Death from direct obstetric cause occurring more than 42 days but less than one year after delivery	400	200
15	Informed consent for procedure	400	300
16	Death from indirect obstetric cause occurring more than 42 days but less than one year after delivery	400	200
17	Suspected fetal damage from radiation	300	200
18	Slow feeding in newborn	300	200
19	Fetal exposure to alcohol	300	200
20	Failed instrumental delivery	300	200
21	Death from sequela of indirect maternal cause	300	200
22	Maternal infection	300	200
23	Adult care	300	300
24	Decolonization	300	300
25	Death from sequela of direct obstetric cause	300	200

Query executed in 3.74 secs

Table 18. Top 25 of mapped procedures. Counts are rounded up to the nearest hundred.

#	Concept Name	#Records	#Subjects
1	Dialysis procedure	412,800	93,900
2	Artificial respiration	400,000	100,000
3	Agreeing on care plan	192,100	92,700
4	Agreeing on diabetes care plan	111,500	73,000
5	Jaw and temporomandibular joint operations	81,500	59,600
6	Prosthodontic procedure	80,600	59,300
7	Orthodontic service	79,900	59,000
8	Periodontic procedure	79,900	59,100
9	Endodontic procedure	79,800	59,100
10	Agreeing on mental health care plan	32,200	28,400
11	Asthma action care planning	31,800	28,300
12	Management of chronic obstructive pulmonary disease	31,700	28,200
13	Hand tendon operation	29,900	15,900
14	Open reduction and fixation	27,700	14,500
15	Reduction procedure	27,400	14,300
16	Open reduction of fracture and external fixation	25,800	13,600
17	Division of musculoskeletal system	24,100	13,200
18	Musculoskeletal system incision	20,100	10,900

#	Concept Name	#Records	#Subjects
19	Excision arthroplasty of joint of hand	18,800	10,300
20	Tenorrhaphy	18,000	10,100
21	Spondylolisthesis operation	17,300	10,100
22	Repair of fascia	16,100	9,200
23	Procedure on bone of forearm	14,700	8,000
24	Trunk repair	14,400	8,400
25	Closed reduction of fracture and external fixation	14,200	7,800

Query executed in 13.56 secs

Table 19 omitted because no mapped devices were found.

Query executed in 0.14 secs

Table 20. All 5 mapped visits. Counts are rounded up to the nearest hundred.

#	Concept Name	#Records	#Subjects
1	Ambulatory Dental Clinic / Center	400,000	100,000
2	Inpatient Hospital	396,000	100,000
3	Outpatient Visit	330,200	87,200
4	Inpatient Visit	82,700	34,500
5	Outpatient Hospital	4,100	4,100

Query executed in 2.40 secs

4.7 Source to concept map

Query executed in 0.14 secs

Note that the full source_to_concept_map table is added in the results.zip

5 Technical Infrastructure

5.1 CDM Source Table

Table 22. cdm_source table content

field	
CDM_SOURCE_NAME	HDL_format3
CDM_SOURCE_ABBREVIATION	MEVIS_format3
CDM_HOLDER	MEVIS
SOURCE_DESCRIPTION	HDL _format3
SOURCE_DOCUMENTATION_REFERENCE	https://github.com/FDZ-Gesundheit/datensatzbeschreibung_fdz_gesundheit
CDM_ETL_REFERENCE	https://github.com/FraunhoferMEVIS/ETLfromHDLtoOMOP/
SOURCE_RELEASE_DATE	2024-06-04
CDM_RELEASE_DATE	2024-09-13
CDM_VERSION	v5.4
CDM_VERSION_CONCEPT_ID	756265
VOCABULARY_VERSION	v5.0 23-JAN-23

5.2 HADES packages

Table 23. Versions of all installed HADES R packages

Package	Version
DatabaseConnector	6.1.0
ParallelLogger	3.3.0
ROhdsiWebApi	1.3.3
SqlRender	1.15.0

Missing HADES packages: CohortMethod, SelfControlledCaseSeries, SelfControlledCohort, EvidenceSynthesis, PatientLevelPrediction, EnsemblePatientLevelPrediction, Capr, CirceR, CohortGenerator, PhenotypeLibrary, EmpiricalCalibration, MethodEvaluation, CohortDiagnostics, Andromeda, BigKnn, Cyclops, Eunomia, FeatureExtraction, Hydra, OhdsiSharing

5.3 System Information

Installed R version: R version 4.2.3 (2023-03-15 ucrt)

System CPU vendor: GenuineIntel

System CPU model: 11th Gen Intel(R) Core(TM) i7-1165G7 @ 2.80GHz

System CPU number of cores: 8

System RAM: 34.07 GB

DBMS: postgresql

WebAPI version: unknown

5.4 Vocabulary Query Performance

The number of 'Maps To' relations is equal to 4372361. This query was executed in 23.26 secs

5.5 Achilles Query Performance

Table 24. Execution time of queries of the Achilles R-Package

ID	NAME	DURATION
0	Source name	0.27
1	Number of persons	0.06
2	Number of persons by gender	0.16
3	Number of persons by year of birth	0.2
4	Number of persons by race	0.17
5	Number of persons by ethnicity	0.15
7	Number of persons with invalid provider_id	0.07
8	Number of persons with invalid location_id	0.08
9	Number of persons with invalid care_site_id	0.07
10	Number of all persons by year of birth by gender	0.17
11	Number of non-deceased persons by year of birth by gender	0.2
12	Number of persons by race and ethnicity	0.13
101	Number of persons by age, with age at first observation period	0.69
102	Number of persons by gender by age, with age at first observation period	0.38
108	Number of persons by length of observation period, in 30d increments	0.49
109	Number of persons with continuous observation in each year	0.65
110	Number of persons with continuous observation in each month	0.61

ID	NAME	DURATION
111	Number of persons by observation period start month	0.21
112	Number of persons by observation period end month	0.21
113	Number of persons by number of observation periods	0.17
114	Number of persons with observation period before year-of-birth	0.2
115	Number of persons with observation period end < observation period start	0.05
116	Number of persons with at least one day of observation in each year by gender and age decile	0.72
117	Number of persons with at least one day of observation in each month	4.75
118	Number of observation periods with invalid person_id	0.14
119	Number of observation period records by period_type_concept_id	0.06
200	Number of persons with at least one visit occurrence, by visit_concept_id	2.44
201	Number of visit occurrence records, by visit_concept_id	0.8
202	Number of persons by visit occurrence start month, by visit_concept_id	3.37
204	Number of persons with at least one visit occurrence, by visit_concept_id by calendar year by gender by age decile	5.77
207	Number of visit records with invalid person_id	0.29
209	Number of visit records with invalid care_site_id	1.51
210	Number of visit_occurrence records outside a valid observation period	0.54
211	Number of visit records with end date < start date	0.17
212	Number of persons with at least one visit occurrence, by calendar year by gender by age decile	4.11
220	Number of visit occurrence records by visit occurrence start month	1.26
221	Number of persons by visit start year	2.18
225	Number of visit_occurrence records by visit_source_concept_id	0.83
230	Number of visit_occurrence records inside valid observation period	0.56
231	Proportion of people with at least one visit_occurrence record outside a valid observation period	1.48
232	Proportion of visit_occurrence records outside a valid observation period	0.65
300	Number of providers	0.77
301	Number of providers by specialty concept_id	1.62
303	Number of providers records by specialty_concept_id and visit_concept_id	2.82
325	Number of provider records by specialty_source_concept_id	0.27
400	Number of persons with at least one condition occurrence, by condition_concept_id	5.75
401	Number of condition occurrence records, by condition_concept_id	1.8
402	Number of persons by condition occurrence start month, by condition_concept_id	9.47
404	Number of persons with at least one condition occurrence, by condition_concept_id by calendar year by gender by age decile	14.42
405	Number of condition occurrence records, by condition_concept_id by condition_type_concept_id	2.04
409	Number of condition occurrence records with invalid person_id	0.59
410	Number of condition occurrence records outside valid observation period	1.18
411	Number of condition occurrence records with end date < start date	0.35
412	Number of condition occurrence records with invalid provider_id	2.81
413	Number of condition occurrence records with invalid visit_id	3.56
414	Number of condition occurrence records, by condition_status_concept_id	1.74
415	Number of condition occurrence records, by condition_type_concept_id	1.73
416	Number of condition occurrence records, by condition_status_concept_id, condition_type_concept_id	1.78

ID	NAME	DURATION
420	Number of condition occurrence records by condition occurrence start month	2.98
425	Number of condition_occurrence records by condition_source_concept_id	1.88
424	Number of distinct people with co-occurring condition_occurrence condition_concept_id pairs	5.11
430	Number of condition_occurrence records inside a valid observation period	1.91
431	Proportion of people with at least one condition_occurrence record outside a valid observation period	4.74
432	Proportion of condition_occurrence records outside a valid observation period	1.38
500	Number of persons with death, by cause_concept_id	0.07
501	Number of records of death, by cause_concept_id	0.07
502	Number of persons by death month	0.08
504	Number of persons with a death, by calendar year by gender by age decile	0.1
505	Number of death records, by death_type_concept_id	0.13
509	Number of death records with invalid person_id	0.06
510	Number of death records outside valid observation period	0.1
525	Number of death records by cause_source_concept_id	0.04
530	Number of death records inside a valid observation period	0.05
531	Proportion of people with at least one death record outside a valid observation period	0.11
532	Proportion of death records that occur outside a valid observation period	0.18
600	Number of persons with at least one procedure occurrence, by procedure_concept_id	10.19
601	Number of procedure occurrence records, by procedure_concept_id	2.98
602	Number of persons by procedure occurrence start month, by procedure_concept_id	19.86
604	Number of persons with at least one procedure occurrence, by procedure_concept_id by calendar year by gender by age decile	28.71
605	Number of procedure occurrence records, by procedure_concept_id by procedure_type_concept_id	3.57
609	Number of procedure occurrence records with invalid person_id	0.94
610	Number of procedure occurrence records outside valid observation period	2.02
612	Number of procedure occurrence records with invalid provider_id	3.38
613	Number of procedure occurrence records with invalid visit_id	4.81
620	Number of procedure occurrence records by procedure occurrence start month	5.88
625	Number of procedure_occurrence records by procedure_source_concept_id	3.67
624	Number of distinct people with co-occurring procedure_occurrence procedure_concept_id pairs	1.44
630	Number of procedure_occurrence records inside a valid observation period	2.38
631	Proportion of people with at least one procedure_occurrence record outside a valid observation period	8.59
632	Proportion of procedure_occurrence records outside a valid observation period	2.53
691	Percentage of total persons that have at least x procedures	7.26
700	Number of persons with at least one drug exposure, by drug_concept_id	1.9
701	Number of drug exposure records, by drug_concept_id	0.82
702	Number of persons by drug exposure start month, by drug_concept_id	3.3

ID	NAME	DURATION
704	Number of persons with at least one drug exposure, by drug_concept_id by calendar year by gender by age decile	4.84
705	Number of drug exposure records, by drug_concept_id by drug_type_concept_id	0.84
709	Number of drug exposure records with invalid person_id	0.27
710	Number of drug exposure records outside valid observation period	0.48
711	Number of drug exposure records with end date < start date	0.17
712	Number of drug exposure records with invalid provider_id	2.37
713	Number of drug exposure records with invalid visit_id	0.17
720	Number of drug exposure records by drug exposure start month	1.25
725	Number of drug_exposure records by drug_source_concept_id	0.72
724	Number of distinct people with co-occurring drug_exposure drug_concept_id pairs	0.8
730	Number of drug_exposure records inside a valid observation period	0.58
731	Proportion of people with at least one drug_exposure record outside a valid observation period	1.62
732	Proportion of drug_exposure records outside a valid observation period	0.62
791	Percentage of total persons that have at least x drug exposures	1.2
800	Number of persons with at least one observation occurrence, by observation_concept_id	7.87
801	Number of observation occurrence records, by observation_concept_id	2.48
802	Number of persons by observation occurrence start month, by observation_concept_id	14.92
804	Number of persons with at least one observation occurrence, by observation_concept_id by calendar year by gender by age decile	22.14
805	Number of observation occurrence records, by observation_concept_id by observation_type_concept_id	2.63
807	Number of observation occurrence records, by observation_concept_id and unit_concept_id	2.59
809	Number of observation records with invalid person_id	0.75
810	Number of observation records outside valid observation period	1.63
812	Number of observation records with invalid provider_id	1.72
813	Number of observation records with invalid visit_id	4.51
814	Number of observation records with no value (numeric, string, or concept)	0.7
820	Number of observation records by observation start month	4.41
822	Number of observation records, by observation_concept_id and value_as_concept_id	2.55
823	Number of observation records, by observation_concept_id and qualifier_concept_id	2.64
824	Number of distinct people with co-occurring observation observation_concept_id pairs	7.55
825	Number of observation records by observation_source_concept_id	2.54
826	Number of observation records by value_as_concept_id	2.64
827	Number of observation records by unit_concept_id	2.57
830	Number of observation records inside a valid observation period	2.01
831	Proportion of people with at least one observation record outside a valid observation period	6.42
832	Proportion of observation records outside a valid observation period	2
891	Percentage of total persons that have at least x observations	5.02
900	Number of persons with at least one drug era, by drug_concept_id	0.07

ID	NAME	DURATION
901	Number of drug era records, by drug_concept_id	0.03
902	Number of persons by drug era start month, by drug_concept_id	0.03
904	Number of persons with at least one drug era, by drug_concept_id by calendar year by gender by age decile	0.03
908	Number of drug eras without valid person	0.04
910	Number of drug_era records outside valid observation period	0.05
911	Number of drug eras with end date < start date	0.04
920	Number of drug era records by drug era start month	0.04
930	Number of drug_era records inside a valid observation period	0.04
931	Proportion of people with at least one drug_era record outside a valid observation period	0.04
932	Proportion of drug_era records outside a valid observation period	0.04
1,000	Number of persons with at least one condition era, by condition_concept_id	0.06
1,001	Number of condition era records, by condition_concept_id	0.04
1,002	Number of persons by condition era start month, by condition_concept_id	0.04
1,004	Number of persons with at least one condition era, by condition_concept_id by calendar year by gender by age decile	0.04
1,008	Number of condition eras without valid person	0.04
1,010	Number of condition_era records outside a valid observation period	0.04
1,011	Number of condition eras with end date < start date	0.04
1,020	Number of condition era records by condition era start month	0.04
1,030	Number of condition_era records inside a valid observation period	0.04
1,031	Proportion of people with at least one condition_era record outside a valid observation period	0.04
1,032	Proportion of condition_era records outside a valid observation period	0.05
1,100	Number of persons by location 3-digit zip	0.33
1,101	Number of persons by location state	0.03
1,102	Number of care sites by location 3-digit zip	0.26
1,103	Number of care sites by location state	0.11
1,200	Number of persons by place of service	0.08
1,201	Number of visits by place of service	1.62
1,202	Number of care sites by place of service	0.25
1,203	Number of visits by place of service discharge type	0.37
1,300	Number of persons with at least one visit detail, by visit_detail_concept_id	0.07
1,301	Number of visit detail records, by visit_detail_concept_id	0.03
1,302	Number of persons by visit detail start month, by visit_detail_concept_id	0.03
1,304	Number of persons with at least one visit detail, by visit_detail_concept_id by calendar year by gender by age decile	0.04
1,307	Number of visit records with invalid person_id	0.04
1,309	Number of visit_detail records with invalid care_site_id	0.04
1,310	Number of visit_detail records outside a valid observation period	0.04
1,311	Number of visit_detail records with end date < start date	0.04
1,312	Number of persons with at least one visit detail, by calendar year by gender by age decile	0.05
1,320	Number of visit detail records by visit detail start month	0.04
1,321	Number of persons by visit start year	0.03
1,325	Number of visit_detail records by visit_detail_source_concept_id	0.03
1,326	Number of records by domain by visit_detail_concept_id	4.6
1,330	Number of visit_detail records inside a valid observation period	0.05

ID	NAME	DURATION
1,331	Proportion of people with at least one visit_detail record outside a valid observation period	0.05
1,332	Proportion of visit_detail records outside a valid observation period	0.04
1,408	Number of persons by length of payer plan period, in 30d increments	0.56
1,409	Number of persons with continuous payer plan in each year	0.51
1,410	Number of persons with continuous payer plan in each month	0.87
1,411	Number of persons by payer plan period start month	0.53
1,412	Number of persons by payer plan period end month	0.51
1,413	Number of persons by number of payer plan periods	0.23
1,414	Number of persons with payer plan period before year-of-birth	0.26
1,415	Number of persons with payer plan period end < payer plan period start	0.13
1,425	Number of payer_plan_period records by payer_source_concept_id	0.32
1,610	Number of records by revenue_code_concept_id	0.3
1,800	Number of persons with at least one measurement occurrence, by measurement_concept_id	0.06
1,801	Number of measurement occurrence records, by measurement_concept_id	0.08
1,802	Number of persons by measurement occurrence start month, by measurement_concept_id	0.06
1,804	Number of persons with at least one mesurement occurrence, by measurement_concept_id by calendar year by gender by age decile	0.16
1,805	Number of measurement occurrence records, by measurement_concept_id by measurement_type_concept_id	0.07
1,807	Number of measurement occurrence records, by measurement_concept_id and unit_concept_id	0.1
1,809	Number of measurement records with invalid person_id	0.14
1,810	Number of measurement records outside valid observation period	0.14
1,811	Number of measurement records with a valid value (with a mapped, non-null value_as_number)	0.04
1,812	Number of measurement records with invalid provider_id	0.05
1,813	Number of measurement records with invalid visit_id	0.28
1,814	Number of measurement records with no value (numeric, string, or concept)	0.11
1,818	Number of measurement records below/within/above normal range, by measurement_concept_id and unit_concept_id	0.08
1,819	Number of measurement records, by measurement_concept_id, with a valid value (with a mapped, non-null value_as_number)	0.04
1,820	Number of measurement records by measurement start month	0.11
1,821	Number of measurement records with no numeric value	0.05
1,822	Number of measurement records, by measurement_concept_id and value_as_concept_id	0.08
1,823	Number of measurement records, by measurement_concept_id and operator_concept_id	0.09
1,824	Number of distinct people with co-occurring measurement measurement_concept_id pairs	0.07
1,825	Number of measurement records by measurement_source_concept_id	0.08
1,826	Number of measurement records by value_as_concept_id	0.08
1,827	Number of measurement records by unit_concept_id	0.05
1,830	Number of visit_detail records inside a valid observation period	0.06
1,831	Proportion of people with at least one measurement record outside a valid observation period	0.12
1,832	Proportion of measurement records outside a valid observation period	0.12

ID	NAME	DURATION
1,833	Proportion of measurement records inside a valid observation period and without a value	0.09
1,891	Percentage of total persons that have at least x measurements	0.08
1,900	Source values mapped to concept_id 0 by table, by column, by source_value	1.87
2,000	Number of patients with at least 1 Dx and 1 Rx	4.23
2,001	Number of patients with at least 1 Dx and 1 Proc	10.39
2,002	Number of patients with at least 1 Meas, 1 Dx and 1 Rx	4.04
2,003	Number of patients with at least 1 Visit	1.19
2,004	Number of distinct patients that overlap between specific domains	35.23
2,100	Number of persons with at least one device exposure, by device_concept_id	0.06
2,101	Number of device exposure records, by device_concept_id	0.03
2,102	Number of persons by device records start month, by device_concept_id	0.05
2,104	Number of persons with at least one device exposure, by device_concept_id by calendar year by gender by age decile	0.05
2,105	Number of device exposure records, by device_concept_id by device_type_concept_id	0.03
2,110	Number of device_exposure records outside valid observation period	0.04
2,125	Number of device_exposure records by device_source_concept_id	0.04
2,130	Number of device_exposure records inside a valid observation period	0.04
2,131	Proportion of people with at least one device_exposure record outside a valid observation period	0.04
2,132	Proportion of device_exposure records outside a valid observation period	0.05
2,191	Percentage of total persons that have at least x device exposures	0.05
2,200	Number of persons with at least one note by note_type_concept_id	0.13
2,201	Number of note records, by note_type_concept_id	0.06

Query executed in 0.34 secs
